# When DNA-damage responses meet innate and adaptive immunity

**DOI:** 10.1007/s00018-024-05214-2

**Published:** 2024-04-17

**Authors:** Jie Tong, Jiangwei Song, Wuchao Zhang, Jingbo Zhai, Qingli Guan, Huiqing Wang, Gentao Liu, Chunfu Zheng

**Affiliations:** 1 College of Life Science, Hebei University, Baoding, 071002 China; 2 Institute of Life Science and Green Development, Hebei University, Baoding, 071002 China; 3 Beijing Key Laboratory for Prevention and Control of Infectious Diseases in Livestock and Poultry, Institute of Animal Husbandry and Veterinary Medicine, Beijing Academy of Agriculture and Forestry Sciences, Beijing, 100089 China; 4 College of Veterinary Medicine, Hebei Agricultural University, Baoding, 071000 China; 5 Key Laboratory of Zoonose Prevention and Control at Universities of Inner Mongolia Autonomous Region, Medical College, Inner Mongolia Minzu University, Tongliao, 028000 China; 6 The Affiliated Hospital of Chinese PLA 80th Group Army, Weifang, 261000 China; 7 Department of Pediatrics, West China Second University Hospital, Sichuan University, Chengdu, 610041 China; 8 Department of Oncology, Tenth People’s Hospital Affiliated to Tongji University & Cancer Center, Tongji University School of Medicine, Shanghai, 20000 China; 9 Department of Microbiology, Immunology and Infectious Diseases, University of Calgary, Calgary, AB Canada

**Keywords:** Adaptive immunity, cGAS–STING, DNA-damage response (DDR), IFN, Innate immunity

## Abstract

When cells proliferate, stress on DNA replication or exposure to endogenous or external insults frequently results in DNA damage. DNA-Damage Response (DDR) networks are complex signaling pathways used by multicellular organisms to prevent DNA damage. Depending on the type of broken DNA, the various pathways, Base-Excision Repair (BER), Nucleotide Excision Repair (NER), Mismatch Repair (MMR), Homologous Recombination (HR), Non-Homologous End-Joining (NHEJ), Interstrand Crosslink (ICL) repair, and other direct repair pathways, can be activated separately or in combination to repair DNA damage. To preserve homeostasis, innate and adaptive immune responses are effective defenses against endogenous mutation or invasion by external pathogens. It is interesting to note that new research keeps showing how closely DDR components and the immune system are related. DDR and immunological response are linked by immune effectors such as the cyclic GMP-AMP synthase (cGAS)–Stimulator of Interferon Genes (STING) pathway. These effectors act as sensors of DNA damage-caused immune response. Furthermore, DDR components themselves function in immune responses to trigger the generation of inflammatory cytokines in a cascade or even trigger programmed cell death. Defective DDR components are known to disrupt genomic stability and compromise immunological responses, aggravating immune imbalance and leading to serious diseases such as cancer and autoimmune disorders. This study examines the most recent developments in the interaction between DDR elements and immunological responses. The DDR network’s immune modulators’ dual roles may offer new perspectives on treating infectious disorders linked to DNA damage, including cancer, and on the development of target immunotherapy.

## Introduction

A board interest in immune response defects caused by DNA damage has emerged. An intimate relationship between DNA damage and immune response provides novel insights into understanding the progress of DNA damage-induced diseases. The occurrence of DNA damage frequently causes genomic instability and subsequent severe diseases [1, 2]. Notably, evidence showed that endogenous DNA damage in steady-state occurs at a frequency of ~ 10^1^–10^2^ per cell per day [3, 4]. Nevertheless, the data may rise to 10^4^–10^5^ per cell per day following the exposure of genotoxic stress [5]. Therefore, the intricate pathways have been characterized for identifying and repairing DNA damage, summarized as the DNA-damage response (DDR) [6, 7]. Following the rupture or mismatch of genomic DNA, the methylated or oxidized bases, intra- and interstrand DNA crosslinks, double-strand breaks, and protein-DNA adducts will occur and then systematically elicit the DDR network. Overall, at least seven distinct pathways may be activated solely or combinedly to repair DNA damage depending on the types of broken DNA, which are base-excision repair (BER) [8], nucleotide excision repair (NER) [9], mismatch repair (MMR) [10], homologous recombination (HR) [11], non-homologous end-joining (NHEJ) [12], interstrand crosslink (ICL) repair and other direct repair pathways [13]. Although numerous genes are involved in the multiple complex processes of DDR, some common programs are shared among diverse repair pathways [14]. Firstly, the specific DNA sensor proteins such as MRE11–RAD50–NBS1 (MRN) complex recognize the aberrant DNA and signal to cell cycle checkpoint and DNA-damage checkpoint kinases. These kinases are activated to induce cell-cycle arrest and recruit other DNA-binding proteins to form the DNA repair complex. The local context of the DNA repair complex then facilitates the activating of endogenous DNA ligase, including DNA polymerase β (POLβ), DNA ligase II, and DNA ligase IV, to fix the damaged DNA [14].

The innate immune responses are the first defense against cellular abnormality in mammalian cells [15]. Cellular innate immune responses are commonly initiated by sensing aberrant cytosolic DNA. Following the sensing step, the cytoplasmic receptors/adaptors are activated to induce the downstream signaling transduction. Various kinases are subsequently recruited and promote the transcription of cytokines, including the multifunctional interferons (IFNs) and pro-inflammatory cytokines [16]. These cytokines then induce the expression of immune effector genes or directly activate intrinsic immune cells to eliminate the insults [17]. The adaptive immune response resists foreign pathogens and tumorigenesis in a long-lasting way. The accumulation of tumor-associated DNA damage activates certain innate immune effectors, participating in DDR and thus regulating adaptive antitumor response [18, 19].

This review focuses on the crosstalk between DNA damage and immune response. Identification of the intimate relationship between DDR and immune response will enlighten the potential therapeutic manners for severe diseases, including tumors and infectious diseases.

## Overview of DNA-damage responses

DNA damage originating from endogenous toxic factors occurs through several mechanisms. Abortive DNA topoisomerase I and II activity and random DNA mismatches produced during DNA replication are two physiological mechanisms that may lead to DNA aberrations and DNA strand breaks [14]. DNA-base lesions, another frequent kind of DNA damage, are produced during hydrolytic and non-enzymatic methylations. Byproducts of oxidative respiration, redox-cycling processes, and Fenton reactions generate reactive oxygen molecules that may cause DNA damage [5]. Macrophages and neutrophils in the context of inflammation and infection also create reactive oxygen and nitrogen compounds, assaulting DNA base-pairing, causing base loss and single-strand breaks (SSBs) [20]. Double-strand breaks (DSBs) are also generated when two SSBs emerge in proximity or the DNA-replication machinery comes across an SSB or other lesions [2]. Although DSBs are not as common as other DNA lesions, all types of DNA damage are exceedingly toxic and difficult to heal. Notably, viral or bacterial infections also unexpectedly caused DNA damage by hijacking pivotal cellular signaling pathways.

Ultraviolet light (UV) is the most significant exogenous factor that induces DNA damage [21]. UV-A and UV-B in bright sunshine cause up to 100,000 DNA lesions in an exposed cell in just one hour [14]. Similarly, ionizing radiation (IR), like uranium decay, induces severe DNA damage, including DSBs. Aside from natural radiation, the artificial radioisotopes original from cancer radiotherapies, such as iodine-131 or technetium-99m, provoke DNA damage even in noncancerous tissue [22]. Moreover, genotoxic DNA-damaging chemicals derived from contaminated foods or drinking water byproducts, such as aflatoxins and heterocyclic amines, also threaten genomic DNA integrity.

**Fig. 1:**
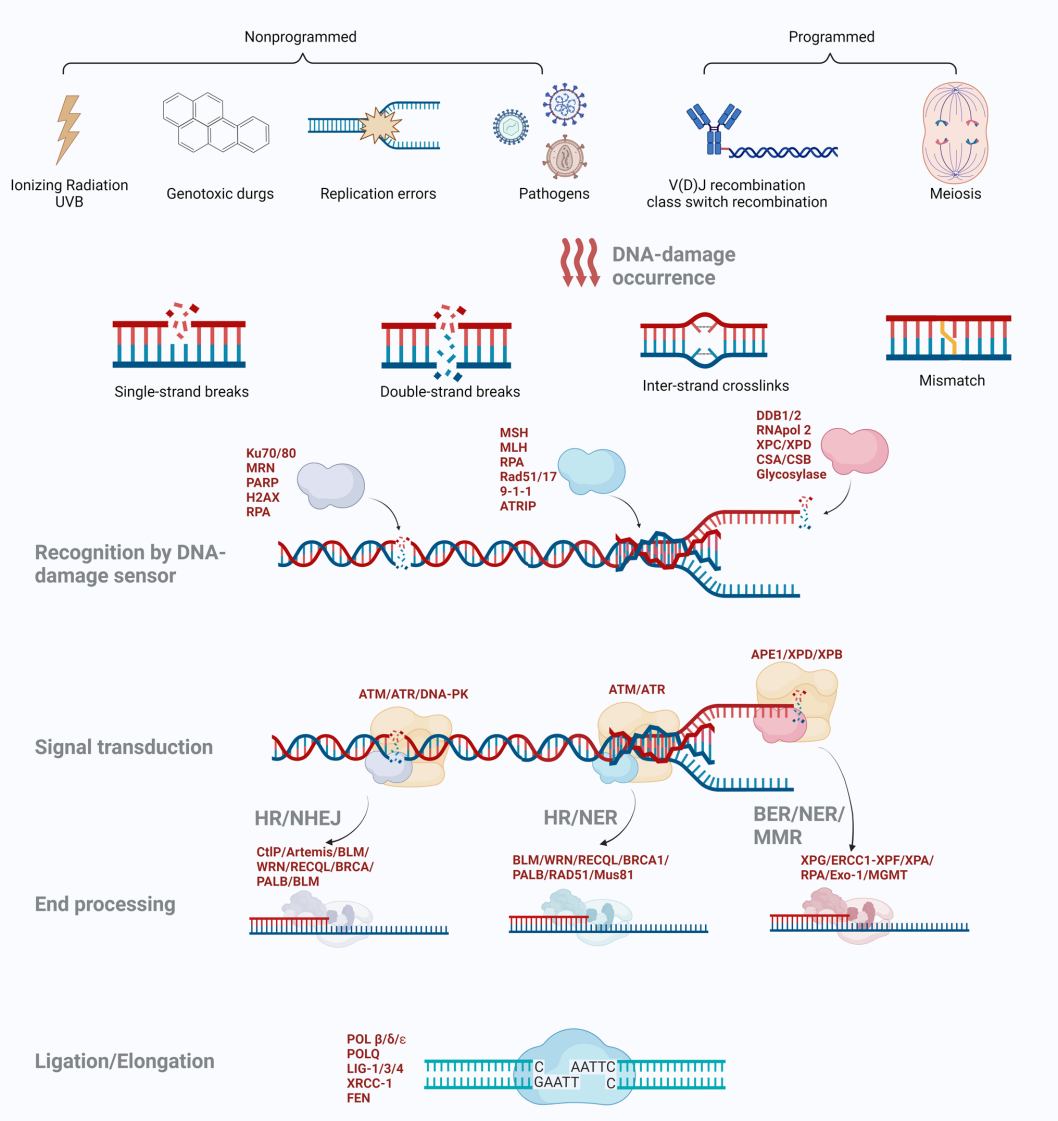
Schematic diagram of DNA-damage response pathways. The integrity of the genome is constantly challenged by genotoxic factors (e.g., inheritance, endogenous reactive oxygen species, DNA replication stresses, or topoisomerase poisons) or exogenous insults (e.g., genotoxic drugs, irradiation, environmental pollutions, or pathogens invasion) that inducing DNA-damage. Accumulation of damaged DNA consequently induces genomic instability. The intricate pathways have been characterized for identifying and repairing DNA damage, summarized as the DNA-damage response (DDR). Overall, at least six distinct pathways may be activated solely or combinedly to repair DNA damage depending on the types of broken DNA, which are base-excision repair (BER), nucleotide excision repair (NER), mismatch repair (MMR), double-strand breaks (DSBs) repair including homologous recombination (HR) and non-homologous end-joining (NHEJ), interstrand crosslink (ICL) repair and direct repair pathways. Intriguingly, although numerous genes are involved in the multiple complex processes of DDR, some common schedules are shared among diverse repair pathways

Therefore, the timely and effective repair of broken DNA is indispensable in maintaining genome stability. Cells have evolved DDR networks to identify and repair DNA lesions (Fig. 1). The BER and NER pathways participate in the repair of single-strand DNA damage. The HR, NHEJ, and FA pathways function in both single-strand and double-strand DNA repair. Despite the powerful and sophisticated DDR network, abnormalities in DDR systems occur occasionally and lead to many severe diseases. Here, the known details of DDR pathways are briefly described below.

### DDR of single-strand DNA break

#### Base-excision repair (BER)

Single-strand DNA damage from mismatch and insertion/deletion loops activate exquisite single-strand repair. The BER fixes the single-strand DNA damage with none or a slightly distorted DNA helix [23–25]. BER pathway mainly responds to oxidized and alkylated DNA bases [26]. Although the extent of damage that triggers BER remains debated, several “common steps” of BER have been described. Briefly, a damage-specific glycosylase scanning and removing the corrupted base initiate the BER pathway. Following the cutting step, the apurinic–apyrimidinic (AP) site occurs in the damaged strand, giving rise to the binding of AP endonuclease and a phosphoribosyl-lase [8]. These enzymes catalyze removing the damaged nucleotide to generate suitable ends for a DNA polymerase.

Consequently, the DNA polymerase enables the addition of the correct nucleotide to the incision under the guidance of the opposite strand before the DNA ligase finally seals the entire backbone. Notably, the BER contains two distinct sub-pathways: short-patch and long-patch [27]. The activation of the short-patch pathway induces rapid fixation of single-base DNA damage during the G1 phase, and the long-patch pathway performs to install two to eight nucleotides surrounding the AP-site during S or G2, which is more time-consuming than short-patch [28].

Five major components are involved in the BER pathway according to the types of damaged DNA: the DNA glycosylase, the AP endonuclease, the DNA polymerase, the DNA ligase, and the other functional partner proteins. DNA glycosylase is the leading part of the BER pathway that initiates the repair process [29, 30]. At least 11 distinct DNA glycosylases are recognized [31]. The known DNA glycosylase varies in recognizing and consequent handling process of oxidative stress products like 7,8-dihydro-8-oxo-2′-deoxyguanosine (OG). For example, human mutT homolog 1 (MTH1) restrains OG incorporation into the DNA strand by hydrolyzing dOGTP and wiping it out from the nucleotide pool [32]. The mutY DNA glycosylase (MUTYH) and the human 8-oxoguanine DNA *N*-glycosylase 1 (hOGG1) excise inappropriate A in OG:A base-pairs and OG in OG:C base-pairs, respectively [8]. More details of DNA glycosylase are well-demonstrated in previous reviews [8, 31, 33, 34]. Following the scanning and exciting step, the AP endonuclease (for example, the APE1) replaces the DNA glycosylase in the incision and recruits the specific DNA polymerase β (pol β) to fill the gap with the correct nucleotides according to the complementary strand in the damaged sites. The pol β then attracts DNA ligase III (LIG 3) with the help of the X-ray repair cross-complementary gene 1 (XRCC1) scaffold protein to promote the polymerase-ligase interaction. The LIG3 seals the fractured DNA strand and disassociates from the working area afterward. The schematic of the BER pathway and the major components in mammalian cells are shown in Fig. 1.

#### Nucleotide excision repair (NER)

As another strong strategy to repair single-strand DNA damage, the nucleotide excision repair (NER) pathway activates to deal with helix-distorting DNA lesions. Two NER pathways have been identified in mammalian cells: global genomic repair (GGR) and transcription‑coupled repair (TCR) [35]. As shown in Fig. 1, GGR and TCR distinctly activate the NER pathway in transcribed or non-transcribed genomic regions, respectively. The partner protein Cockayne syndrome A (CSA, also known as ERCC8) and CSB (also known as ERCC6) ubiquitylation the C‑terminal domain of RNA polymerase II, which arrested in the damaged coding region and initiated the normal NER through GGR signaling [36, 37]. On the other hand, DNA damage‑binding 1 (DDB1) and DDB2 are recruited in the non-transcribed region to be detected by xeroderma pigmentosum (XP) group E (XPE) in a TCR manner.

The functional components presenting in the normal NER pathway include six core factors. The recognizing and incision process is realized by heterocomplex consisting of the XPA protein, the single-strand DNA binding heterocomplex replication protein A (RPA) [38], the XPC-human Rad23 proteins B(hHR23B) complex [39], the 6–9 subunit TFIIH complex [40], and two nucleases, the XPG and the heterodimeric excision repair cross-complementing gene 1 (ERCC1)-XPF [41]. Afterward, XPB and XPD unwind the DNA helix and allow the XPG and ERCC1-XPF to access the damage site to cut off a 24- to 32-residue oligonucleotide. After that, the DNA polymerase δ or ε (POLδ/ε) and a DNA ligase (LIG1) come across to pad the gap and seal the strand [42]. Although the precise mechanisms of NER recognizing patterns remain vague, accumulating evidence has shown that NER pathways’ components are associated with other cellular signaling pathways, such as the innate immune response pathways. The details of related insights will be further discussed below.

### DDR of double-strand breaks

#### Homologous recombination (HR)

Homologous recombination (HR) is one of the primary DDR pathways for repairing DSBs in DNA. The HR pathway generally works during the S and G2 phases due to the specific template donor for repairing the sister-chromatid sequences. Therefore, the HR repair of DSBs is highly reliable compared to other DDR pathways commonly described as error-free repairing.

Unlike single-strand DNA faulty, repairing DSBs begins with flexible resection of the dsDNA into ssDNA with 3′-OH terminals. The nuclease heterocomplex MRE11-RAD50-NBS1(MRN) facilitates the incision and recruits the ataxia telangiectasia-mutated (ATM) to facilitate it separate from homo-dimers to monomers [43]. ATM belongs to the phosphatidylinositol 3-kinase (PI3K)-like kinase (PIKK) family and activates various downstream cascades. Several intermediate proteins also form repair foci during the initial step through diverse mechanisms. Among them, RAD51 binds to the ssDNA to create right-handed helical filaments, which serve as nucleoprotein scaffolds for the subsequent repair [44]. RAD51 mediates the scanning of the homology template donor and strand invasion of a homologous duplex to form a displacement loop (D-loop) [45].

In contrast, the ssDNA-binding protein RPA competes with RAD51 against the forming filaments. Besides, breast cancer susceptibility proteins (BRCA) 1/2 and some other nuclease may also participate in the resection step [46]. After the ssDNA filaments generation and ATM activation, a cascade of downstream effectors, including DNA helicase RecQ Like Helicase (RECQL) 1/4/5, DNA nuclease bloom helicase (BLM)/Werner protein (WRN) [47], and DNA ligase are activated to execute the strand invasion, DNA ligation, and substrate resolution in a relatively slow rate. Especially, the checkpoint kinase 1/2 (CHK1/2) and histone 2A (H2A) phosphorylation (termed as γH2AX) are also regulated by ATM during the HR process, which inhibited the activation of cyclin-dependent kinase 1 (CDK1) to arrest the cell cycle by regulating S/M phase and blocking G2/M transition [48].

#### Non-homologous end-joining (NHEJ)

Compared to the HR, NHEJ repairs DNA-DSBs more efficiently but imprecisely during the entire cell cycle phase. Complex regulatory mechanisms control the decision between the NHEJ and HR pathways, influenced by the conflict between p53-binding protein 1 (53BP1), which favors NHEJ [49], and BRCA1, which supports HR [50]. The methylation of histone H4 in the DSB site recruits 53BP1, thus preventing the MRN complex, C-terminal binding protein 1 interacting protein (CtIP), and BRCA 1 from cutting DNA ends. The Tip60, on the other hand, prevents 53BP1 recruitment and encourages BRCA1 occupancy to perform HR after histone H4 acetylation [51]. BRCA1 and other proteins that control the cell cycle, such as cyclin-dependent kinases (CDK), are also crucial for determining the best pathway for repairing DNA damage [52].

Although several sub-pathways have been elucidated during the NHEJ process, the common steps are briefly divided into two manners. In the canonical NHEJ (cNHEJ), the collapsed DNA strands are firstly detected by lupus Ku autoantigen protein (Ku) protein (mainly ku70–ku80 heterodimer), which recruits DNA protein kinase C (DNA-PKcs) to form a DNA-PK complex with DNA [53]. DNA PKcs then recruit and activate nuclease Artemis, polynucleotide kinase phosphorylase, and other DNA polymerases to further generate the repairing foci. Then, the XRCC4/DNA ligase IV is stimulated to re-connect the break [54, 55]. The other type of NHEJ is designated as alternative NHEJ (aNHEJ), independently initiating aside from the Ku complex. In addition, the alternative NHEJ pathway takes advantage of the DNA polymerase θ (Pol θ or POLQ), DNA ligase III, XRCC1, and PNK to deal with chromosome abnormalities, including translocations, deletions, and inversions [56].

### DDR of inter-strand crosslinks (ICLs)

Repairing interstrand crosslinks (ICLs) involves another DDR pathway, such as NER and HR. At least twenty-two “FANC” proteins in the Fanconi anemia (FA) family, including BRCA1, regularly start and facilitate the ICL repairing process [57]. The WRN protein may also play a role in stabilizing the structure of DNA replication forks, which is then cleaved by Mus81-EME1 to produce a one-ended double-strand break [58]. DNA opposite is further created by trans-lesion synthesis. The consequent unhooking of the covalently bound crosslink requires NER [59]. During the mitosis of mammalian cells, HR components stabilize and reset the collapsing fork, enabling the DNA replication to continue.

### DDR of mismatch and other types of abnormal DNA

In addition to severe breaks, other faulty DNAs occur due to error-prone DNA polymerases, unstable replication forks, and other endogenous and exogenous assaults. The base pair mismatch, including insertion and deletion during the DNA replication, is generally fixed by the mismatch repair (MMR) pathway, which mainly depends on DNA mismatch repair proteins. Briefly, the heterodimer Mutator Sα (MUTSα, consisting of Mutator S homolog 2 and Mutator S homolog 6) and Mutator Sβ (consisting of Mutator S homolog 2 and Mutator S homolog 3) sense the deletion, insertion, and mismatch site on the DNA strand. Afterward, the Mutator L (MutLα) [MLH1/postmeiotic segregation increased 2 (PMS2)] or MutLβ (MLH1/MLH3) cleave the lesion site. Then, exonuclease 1 (Exo1) decays the mistake nucleotides before the DNA ligase I joins the single-stranded DNA gap [10]. Furthermore, mammalian cells utilize a single-step repair to avoid thymine or guanine alkylation induced by G:C to A:T transitions or strand breaks. The *O*^6^-methylguanine-DNA methyltransferase (MGMT) only cut the alkyl groups from the aberrant nucleotides [60].

### Overview of DDR components: ATM, ATR and DNA-PK

ATM, ATR, and DNA-PK recruitment and activation at the DNA-damage site are the core roles in DDR [61]. Precise regulation of ATM, ATR, and DNA-PKcs is necessary to avoid toxic DNA repair, cell-cycle arrest, aging, and apoptosis due to inappropriate activation. Protein co-factors are essential for the steady recruitment of these three kinases to DNA damage sites; for example, DNA-PKcs need Ku70/80, ATR needs ATRIP, and ATM needs NBS1. Interestingly, NBS1, ATRIP, and Ku80 all share a similar C-terminal motif required for PIKK binding via interactions with the PIKK HEAT repeat. Therefore, a common rule may exist for their recruitment, even if each kinase requires a distinct component.

Remarkedly, the depletion of these three kinases results in the over-activation of immune responses, indicating their modulatory role in the immune signaling pathway. For example, ATM inhibition or depletion commonly results in higher sensitivity to cGAS–STING-mediated IFN-I production in inflammatory disease and tumorigenesis [62, 63]. In line with ATM alone, inhibitors’ attenuation of the ATM/CHK2 axis stimulates STING-dependent immune response in ARID1A-deficient tumors [64]. Additionally, ATR may also play multifaced roles in IFN signaling pathways, either positively regulating the expression of IFN [65] or abolishing the signaling transduction of IFN pathway [66, 67]. Combining ATR and PARP inhibitors potentiates the cell death of ATM-deficient cancer cells, which may benefit immunotherapy treatment [61, 68]. Therefore, the auto-phosphorylase in the DDR network may be the target of antitumor immunotherapy.

## The regulation of innate immune sensors by DDR components

### The cytosolic DNA sensors

#### cGAS

**Fig. 2:**
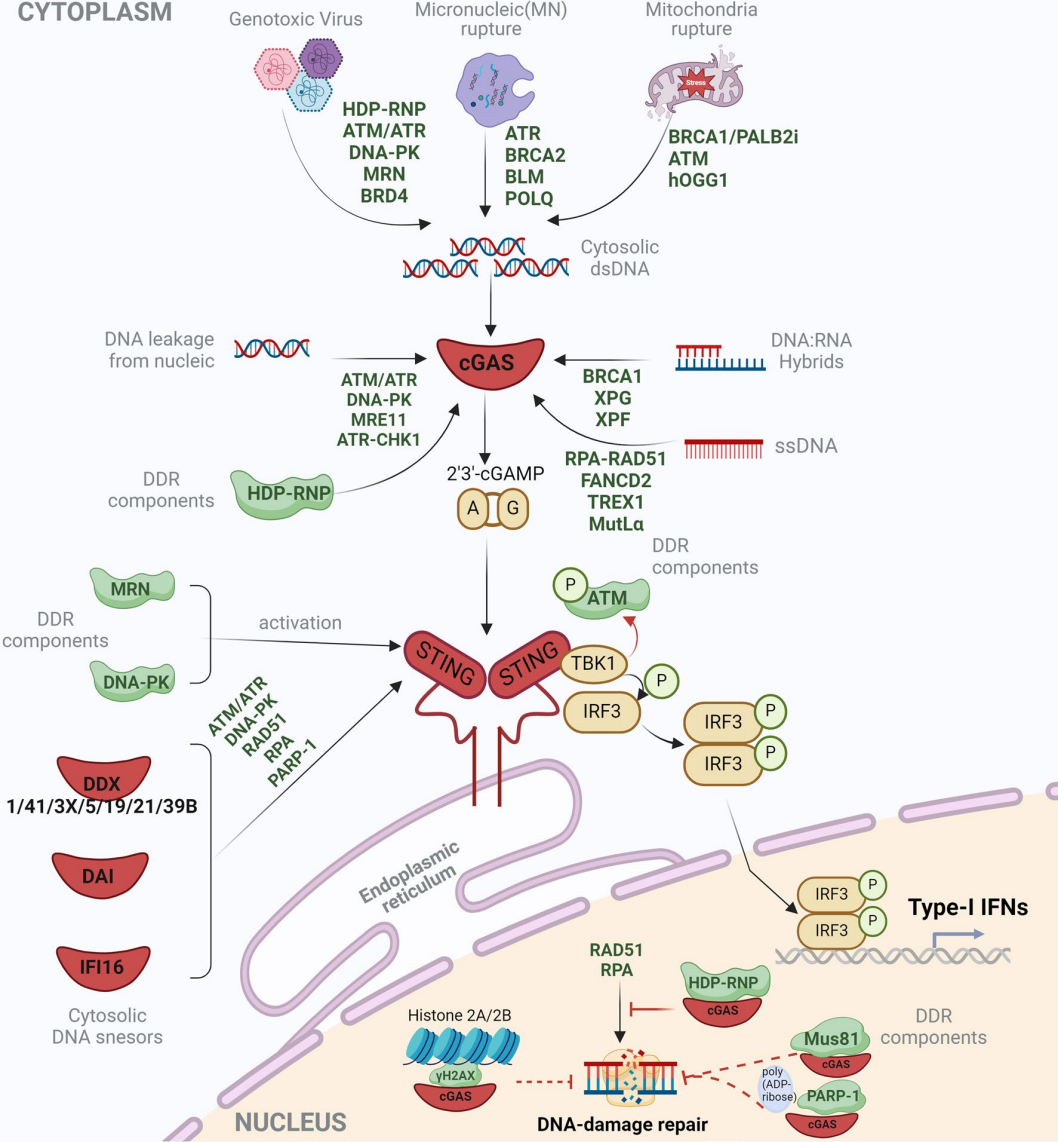
DDR components regulate IFNs signaling pathways. The cellular innate immune response frequently intimately communicates with DDR components via DNA sensors-dependent IFNs signaling pathways. In the IFNs pathways, many DDR components regulate the initial sensing process by interacting with cGAS. cGAS is the universal dsDNA sensor that catalyzes the formation of cGAMP and comprises a heterocomplex with cGAMP to signal the cytosolic adaptor STING activation, which subsequently initiates IFN-1 production, inflammation response, or programmed cell death pathways. Several DDR components also participate in the STING and its downstream signaling pathways. Except for cGAS, other cytosolic DNA sensors like the DDX family, DAI, or IFI16 have also been found to associate with DDR components. Moreover, considering the nuclear location of cGAS, certain DDR components may interact with cGAS inside the cell nucleus, which indicates a potential role of cGAS in regulating the DNA-damage repairing process

cGAS catalyzes the formation of cGAMP and comprises a heterocomplex with cGAMP to signal the cytosolic adaptor STING activation, which subsequently initiates IFN-1 production, inflammation response, or programmed cell death pathways [69, 70]. cGAS responds to endo-/exogenous stress-induced mtDNA or genomic DNA fragments leakage from nucleic and pathogens-derived dsDNA, ssDNA, and RNA–DNA hybrids [71, 72]. Remarkedly, cGAS translocation into the nucleus and response to dsDNA arrested in cytoplasmic micronuclei facilitates its possible crosstalk with DDR components (Fig. 2).

Previous studies indicated that cGAS normally closely couples with histones 2A and 2B to prevent the catalyzing of nucleosomal DNA during cell mitosis [73–75]. Nevertheless, cGAS has been found to directly suppress DNA repair [76, 77], mechanistically in tumorigenesis by directly binding cGAS to DDR components inside the cell nucleus. Following DNA damage, cGAS binds to and forms a complex with Poly-ADP-ribose polymerase 1 (PARP1) [78]. Further research indicated that cGAS enables interaction with another complex composed of the long non-coding RNA NEAT1, the hexamethylene bis-acetamide-inducible protein 1 (HEXIM1), DNA-PK, and paraspeckle factors, which are termed HDP-RNP, inside the nucleus. The interaction between DDR components and cGAS activates the downstream signal of cGAS. Additionally, the activation of cGAS/STING during tumorigenic transformation has also been associated with another fork-processing nuclease, Mus81, in the therapeutic of CINII-stage prostate tumors, suggesting a causal relationship between cGAS sensing and DNA repair [79]. Whereas, as a cytosolic residence, more evidence of intra-nuclear cGAS interacting with DDR components remains to be elucidated.

DDR components mediating cytosolic dsDNA accumulation will inevitably affect the sensing of self- and non-self-DNA by cGAS. For instance, the abrogation of BRCA1-PALB2 interaction in hepatocellular carcinoma (HCC) cells potently induced mtDNA leakage into the cytoplasm to be sensed by cGAS [80]. Therefore, many DDR components are supposed to play negative roles in regulating cGAS-associated innate immune response. In some cases, the negative role of DDR components in regulating cGAS sensing might protect the cells from unfavorable activating of inflammatory responses to self-DNA. For example, the ER-anchored three prime repair exonuclease 1 (TREX1) degrades ssDNA, thereby protecting the cGAS hyperactivation in sensing self-DNA [81]. TREX1 deficiency contributes to autoimmune diseases like Aicardi–Goutières syndrome (AGS) [82].

On the contrary, some DDR components capable of cutting DNA may positively regulate the cGAS sensing process [83]. Aside from dsDNA, the short single-stranded DNA (ssDNA) or RNA–DNA hybrids occurring in cytoplasm induced by RPA-RAD51 knockdown or FANCD2 abrogation also causes IFN-I expression depending on cGAS activation [84, 85]. In HEK293T cells, the sterile alpha motif and HD domain-containing protein 1 (SAMHD1) activate the MRE11 to exert the ATR-CHK1 restarting DNA replication forks, thereby inhibiting the ssDNA fragment from being released from nucleic and sensed by cGAS [86, 87]. RNA–DNA hybrids also act as the substrates of cGAS. Deleting endonuclease XPG and XPF increases the accumulation of R-loop-originated cytoplasmic RNA–DNA hybrids, which ulteriorly stimulate the cGAS activation [88]. BRCA1 plays a similar role in activating the cGAS dependent-IRF3 pathway by regulating the amounts of cytoplasmic RNA–DNA hybrids in HeLa cells [89].

In severe cases of genotoxic virus infection, DDR networks are induced to maintain genomic stability. Therefore, cGAS communicates the DDR to antiviral innate immune response via sensing of virus-derived DNA [90]. Some DNA viruses elicit DDR to arrest the cell cycles to facilitate viral genome synthesis [91]. In other cases, the virus interferes with DDR pathways to induce programmed cell death. The dead cells provide high efficiency of the offspring virus’ release [92–96]. Many other RNA viruses have also been found to impose cytosolic DNA leakage and activate the cGAS–STING pathway afterward. For example, in norovirus-infected cells, host genomic DNA or mtDNA was accumulated in the cytoplasm, which ensures the activation of the cGAS–STING pathway to induce the IFN-β expression [97]. DDR may be the intermediator of virus-induced innate immune responses in these contexts.

Micronuclei maintain cytosolic DNA and serve as the immuno-stimulator of the cGAS/STING pathway under the control of cell-cycle progression [98, 99]. Previous studies implied that the DDR components regulate cGAS activation through micronuclei intermediating. For example, inhibition of ATR by M6620 significantly induces lung cancer cells harboring more than two micronuclei, eliciting cGAS-micronuclei interacting to activate immune responses and partially enhance the responses to immunotherapy [100]. Depletion of ATR [101] or BLM-RECQL also strengthens the cGAS-containing cytoplasmic micronuclei forming, indicating these DDR components might obstruct the cGAS sensing pathway [102]. BRCA2 deficiency induces a high frequency of stalled DNA replication forks that enhance the number of cGAS-positive micronuclei, consequently provoking a cascade of inflammatory cytokine expression [103]. Similar results have been found in DNA polymerase θ (POLQ) knockdown BRCA2-deficient pancreatic ductal adenocarcinoma cells [104]. However, the latest solid research has indicated that the chromatin bridges serve as the direct and strong stimulators of cGAS rather than micronuclei [105]. Therefore, there may exist multiple sub-cellular structures in communicating DDR network to cGAS activation.

#### STING

STING is the cornerstone of cGAS-induced immune signaling pathways [106]. Canonically, cGAS catalyzed the formation of cGAMP and facilitated cGAMP binding to STING. The interaction of cGAMP and STING liberates the carboxyl terminus of STING to recruit and phosphorylate TBK1 and IFN regulatory factor 3 (IRF3). Nuclear input of phosphorylated (pIRF3) enables it to act as the transcriptional activator of IFNs through binding to IFN promoter and induces the expression of IFNs and the subsequent IFN stimulated genes (ISGs) [107]. DDR factors intersect with STING to induce innate immune responses in various cells (Fig. 2). For example, ATM depletion makes cancer cells more sensitive to ATR inhibitors or DNA damage, which ensures high levels of IFN expression dependent on the cGAS–STING signaling pathway. In preclinical models, STING signaling in DCs was triggered by oxidized mtDNA generated from radioactively treated cancer cells employed as vaccines and was essential for inducing antitumor immune responses [108]. Furthermore, cGAS–STING signaling was imperative in immune response activation following ionizing radiation-induced mtDNA leakages [108–111].

In other cases, STING may respond to cytosolic DNA sensors other than cGAS (Fig. 2). For example, the PARP–ATM–IFI16 axis could activate STING to induce IFNs production [112]. Likewise, Mus81 interacts with STING and boosts the expression of IFNs independent of cGAS [79, 113]. Moreover, Manganese chloride induces oxidative DNA damage in neurons and other human cells [114, 115]. STING-dependent IFN-I expression in manganese (Mn) treated HEK cells strongly depends on the interaction between DDR kinase ATM instead of cGAS [116]. Other DDR components that interact with STING include DNA-PK and Ku70 [117, 118]. These evidences imply STING is a crucial mediator in the DDR network.

#### Toll-like receptors (TLRs)

Another group of cytosolic DNA sensors participating in intrinsic immune response has been known as Toll-like receptors (TLRs). TLRs recognize dsDNA and generally activate to elevate inflammation response and programmed cell death, including autophagy against cellular insults. When DNA damage occurs due to oxidative stress, certain components of the DNA damage response (DDR) may influence the expression of TLRs, hence regulating cellular inflammatory responses (Fig. 4). TLR2 stimulates the signaling transduction of myeloid differentiation primary response protein 88 (MyD88) to activate the NF-κB pathway. In breast cancer cells, TLR2 has been found to be associated with the high-mobility group box 1 (HGMB1) to facilitate anti-tumor immune responses [119, 120]. TLR9 is often responsible for detecting dsDNA that is found in endosomes [121]. TLR9 also responds to other specific types of DNA, such as mitochondrial DNA, including damage-associated molecular patterns (DAMPs), leading to the release of IL-8 in human neutrophils. Blocking of OGG1 also enhances the ability of TLR9 to detect mtDNA [122]. Deficiencies in TLR2 and TLR4 also can hinder the efficacy of DDR by decreasing the expression of OGG1 in chondrocytes, suggesting that OGG1 may also play a role in the TLR2/TLR4 signaling pathway [123]. Additionally, PARP-1 functions as the regulatory factor that promotes the activation of TLR4 via restraining the activity of sirtuin 1 (SIRT1) [124–127]. Moreover, knocking down DNA-PKcs impairs the expression of TLR1, TLR3, and TLR8 in RAW264.7 cells via modulating the activity of their promoters [128], indicating an intimate interaction between TLRs and the DDR network.

#### DEAD-box (DDX) RNA helicase family

In mammalian cells, members of the DEAD-box (DDX) RNA helicase family have bound to DNA through their DEAD domain and induce IFNs production via the STING–TBK1–IRF3 pathway [129, 130]. Meanwhile, some DDX proteins also function in preserving genomic DNA integrity and DDR signaling pathways [130, 131]. Although no evidence has shown the direct activation of DDX family proteins in the context of DDR, as crucial cytosolic DNA sensors, DDX41 and DDX1may bind to the micronuclei DNA or DNA: RNA hybrids upon the defective DDR, therefore activate the IFN responses [132–137].

#### Other cytoplasmic DNA sensors

A great deal of work has gone into trying to figure out how DNA damage triggers signaling cascades. One of the possible cytosolic DDR sensors was identified as a DNA-dependent activator of IRFs (DAI, sometimes referred to as Z-DNA binding protein, ZBP-1). DAI binds to several types of cytoplasmic DNA to activate IFNs production via the TBK1–IRF3 axis, which makes it necessary for the response of endogenous aberrant DNA [138]. Significantly, a recent study showed that ZBP-1-RIPK3 signaling that caused T cell necroptosis is stimulated by knocking down RPA1, suggesting that ZBP-1 may function as a positive sensor for DNA damage [139]. Retinal pigment epithelial cells consistently expressed significant levels of ZBP-1 in response to oxidative stress-induced mtDNA accumulation [140]. The ZBP1-MLKL necroptotic cascade in radiation-damaged tumor cells caused cytoplasmic DNA to accumulate, which in turn stimulated the cGAS–STING signaling pathway, creating a positive feedback loop that perpetuates inflammation [141]. Numerous studies have suggested that various cytosolic DNA sensors convey DNA damage information to immune responses that rely on complex mechanisms.

### The nuclear DNA sensors

DNA damage and the ensuing DDR in the nucleus may increase the likelihood that DDR components will encounter nuclear-resident immune sensors. Nonetheless, a small body of work has shown that nuclear DNA sensors react to damage to genomic DNA. cGAS is one of the important immunological sensors found in the cell nucleus. Histones 2A and 2B can be bound by cGAS during cell mitosis, as previously mentioned. On the other hand, cGAS will be triggered and cause signal transduction of the STING–IRF3–IFN pathway if the histone DNA is fragmented or damaged. The cGAS activation in this scene has been found to be regulated by PARP, HDP-RNP, and Mus81.

#### Absent in melanoma 2-like receptors (ALRs)

By detecting dsDNA in the cytoplasm of cells, the pyrin and HIN domain-containing missing in melanoma 2 (AIM2) protein functions as a potent activator of the inflammasome to trigger IL-1β and IL-18 secretions. It has been discovered that AIM2 directly detects DSBs within the nucleus to cause intestinal epithelial cells and bone marrow cells to die in a caspase-1-dependent manner [142, 143]. Because of their structural similarities, several proteins in the AIM2-like receptor (ALR) family are assumed to serve as frequent intracellular DNA sensors that initiate the innate immune response. Due to the better effectiveness of DNA-damage response, mice and cells lacking ALRs are more resistant to the genotoxic effects of chemotherapy and ionizing radiation [142, 144]. Nuclear ALRs attach to chromatin, preventing DNA repair machinery from reaching the damaged location and encouraging self-oligomerization, which compacts the chromatin [144]. ALRs may be a therapeutic target for illnesses caused by DNA damage because they may interact more directly than other DNA sensors with DDR components, particularly in the nucleus [145]. Additionally, by activating caspase 1 and cleaving pro-IL-1β and pro-IL-18 to generate IL-1β and IL-18, respectively, ATM and DNA-PKcs support AIM2 inflammasome activation [146, 147]. In fact, because ATM dysfunction causes improper inflammasome production, it compromises innate immune responses [148].

#### Interferon inducible protein 16 (IFI16)

IFI16 is another nuclear DNA sensor that reacts to damage to DNA. IFI16 is a member of the PYHIN family, which includes other members that are involved in DDR pathways and cell-cycle regulation. IFI16 is largely recognized as an immune stimulator by nuclei sequence-independently inducing the inflammasome pathways by recognizing virally generated dsDNA [149]. DNA-PK directly phosphorylates IFI16 at T149 in HSV-1-infected fibroblasts, and both phosphorylation events combined trigger the production of IFN-β [150, 151]. However, it is yet unclear if DNA-PK influences IFI16’s ability to detect viral DNA. It is noteworthy that IFI16 is thought to respond to genomic DNA damage by downregulating DDR components and triggering the production of IFNs or cytokines. With IFI16, both ATM and PARP-1 are connected. Following etoposide therapy, ATM stimulates the binding of IFI16 to p53 and additionally encourages the heterodimer translocation into the cytoplasm, hence inducing the production of STING-dependent cytokines [112]. It was demonstrated that PARP-1 improves the IFI16-p53 interaction [152]. Additionally, as the downstream signal of ATM-dependent cell-cycle regulation, the IFI16-p53 complex interacts with BRCA-1 [153]. As with IFI16, affinity purification-mass spectrometry analysis links IFIX to DDR components [154]. After being exposed to ionizing radiation, IFIX is exported from cell nuclei and may help to activate immune responses [155]. Consequently, by directly controlling DDR components, IFI16 or IFIX may act as a mediator between immunological responses and DDR signaling pathways.

### DDR components directly sensing DNA

The initial stage in DDR is typically to recognize and bind to the DNA damage site. Sequence-independent mechanisms allow DNA-damage sensors to directly initiate innate immune signaling pathways either inside or outside of nuclei.

#### MRN

MRE11 can function as a sensor to trigger innate immune responses in addition to sensing DSBs. Together with RAD50 and NBS11, MRE11 forms an MRN complex that binds to cytosolic dsDNA and triggers STING-dependent IFN-I expression [156]. Notably, RAD50 directly interacts with CARD9 to promote pro-IL-1β production and NF-κB activation in DCs, highlighting RAD50’s function in immune responses [157]. Furthermore, MRE11 was solely shown to sense mtDNA [158] and viral genomic DNA, such as mice’s autonomous parvovirus minute virus [159].

#### DNA-PK

Similarly, when the DNA-PK complex, made up of Ku70/80 and DNA-PKcs, binds to cytosolic dsDNA, comprising self-DNA and DNA originating from microbes, downstream STING-TBK1 is activated to produce IFN-I and inflammatory cytokines [160–162]. In particular, Ku70 translocates into the cytoplasm, where it senses DNA from cytosolic viruses to trigger the production of IFN-λ1, a Type-III IFN that IRF-1 and IRF-7 mediate against viral infection [91, 117, 118]. It has been proposed that STING functions as a downstream adaptor for the Ku complex, which phosphorylates IRF3 to trigger IFN transcription [117, 145]. Interestingly, DNA-PK stimulates IFN-β production through the SIDSp pathway, which is independent of STING. DNA-PK detects the terminal of transfected or virus-derived dsDNA, in contrast to the patterns of cGAS sensing [161].

### The cytosolic RNA sensors

#### RNA polymerase III

**Fig. 3:**
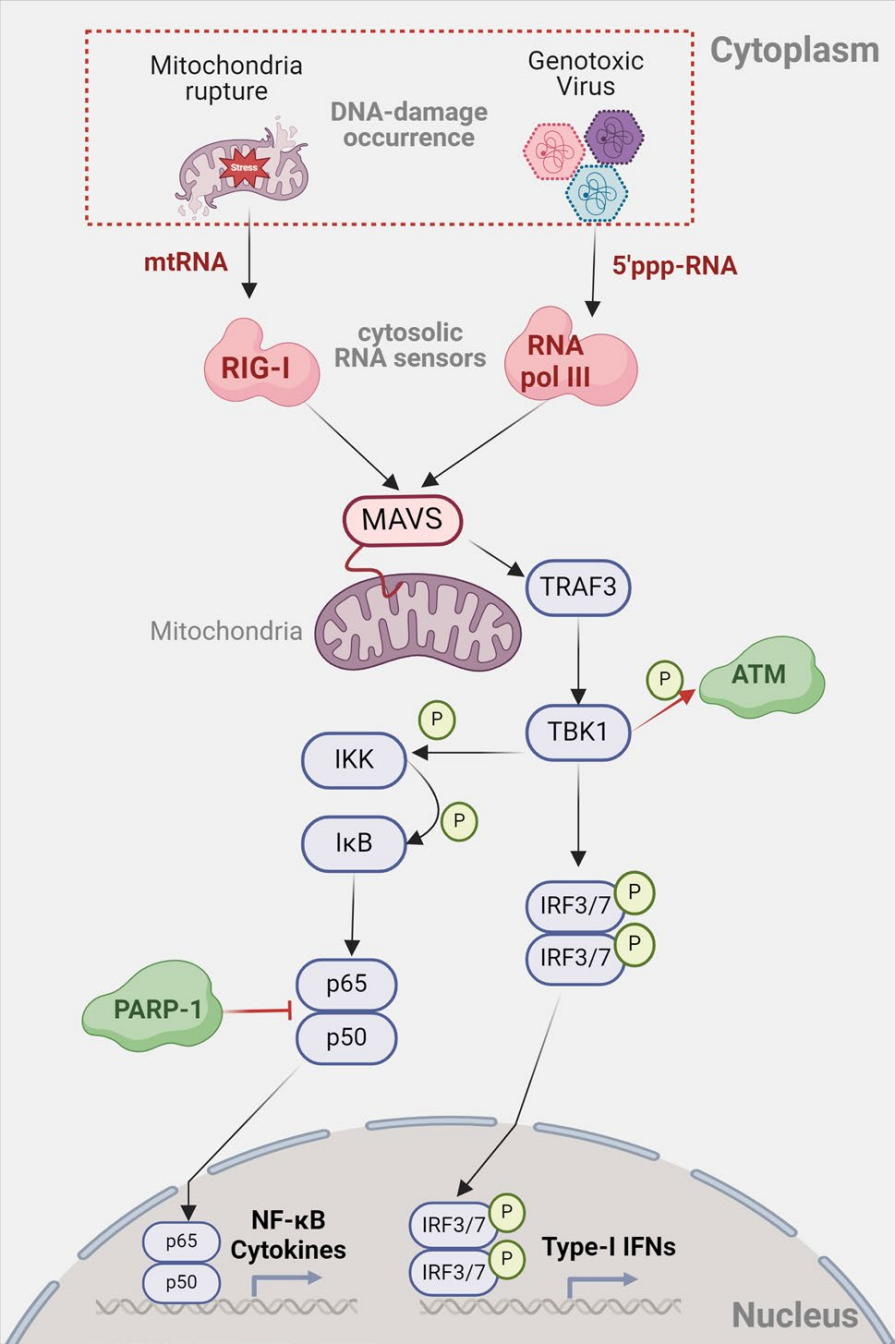
DDR components regulate cytosolic RNA sensors-dependent IFNs and NF-κB signaling pathways. The cytosolic RNA sensors (e.g., RIG-I and MDA5) signal to MAVS (also known as IPS-1, VISA, and Cardif) and induce IFN-I production or NF-κB activation through the TBK1-IRF3/7 signaling pathway. To date, The cellular innate immune responses also have been implicated under the control of DDR components via RNA sensors-dependent IFNs or NF-κB signaling pathways. For example, the RIG-I or RNA polymerase III responds to mtRNA or viral-derived 5′-pppRNA in the context of DNA damage

It was previously established that RNA polymerase III, or RNA pol III, is in charge of the transcription of short non-coding RNA, such as U6 snRNA and tRNAs. According to recent research, DNA: RNA hybrids are formed at the DSB site during the HR process by RNA pol III synthesizing the complementary RNA strand to the 3′-end redundancy of broken DNA strands [163]. Interestingly, RNA pol III’s cytosolic version may function as RIG-I’s essential patterner to trigger innate immune responses against viruses. RNA pol III initially identifies AT-rich ssDNA from DNA viruses or bacteria mechanically. RNA pol III then initiates the transcription process to synthesize 5′-pppRNA (Fig. 3). RIG-I then seems to identify the 5′-pppRNA (AU-rich), which causes the RIG-I–MAVS–TBK1–IRF3 pathway to induce the production of IFN-β [164]. In several DNA virus-infected cells, as well as possibly in RNA virus-infected cells, RNA pol III has been shown to take part in the antiviral innate immune response [165]. Thus, in the context of viral infection, RNA pol III might serve as an active mediator between DDR and the antiviral immune response.

#### Retinoic acid-inducible gene I (RIG-I)-like receptors

Cytosolic dsRNA or ssRNA is largely sensed by receptors that resemble RIG-I (retinoic acid-inducible gene I). Through the TRAF3–TBK1–IRF3 signaling pathway, RIG-I and MDA5 signal to MAVS (also known as IPS-1, VISA, and Cardif) to induce the production of IFN-I. It has recently come to light that ionizing radiation and chemotherapy damage mitochondrial DNA, leading to double-strand breaks (mtDSBs) and herniations that result in the release of mitochondrial RNA (mtRNA) into the cytoplasm. Subsequent accumulation of mtRNA triggers an immunological response that is dependent on RIG-I and MAVS [166]. The most recent work has consistently shown that the RIG-I–MAVS signaling pathway-driven mtRNA leaking is caused by the chemotherapeutic medication cisplatin [167]. These data imply that when DNA is damaged, innate immune signaling is triggered via RNA sensing pathways. As a result, the sensors of mtRNA and cytosolic RNA are crucial mediators between the immune system and DNA damage (Fig. 3).

## The interactions between innate immune signaling pathways and DDR network

Upon the DNA damage, diverse physiological responses aside from DDR will be onset both in the nucleus and cytoplasm, including immune signal transduction, cell-cycle arrest, systematic inflammations, and even programmed cell death. Among them, innate immune responses are major effectors of DNA damage, which also tightly connect to other physiological processes. Demonstrating the sophisticated effectors of DNA-damage-induced immune responses may deepen our understanding of etiological factors in various diseases.

### TBK1–IRF3 signaling axis

TBK1 is a multifaced serine/threonine kinase protein that is a member of the IKK family and is essential to the pathway that produces IFN. TBK1 can directly initiate ATM autophosphorylation and trigger the subsequent DDR activation in THP1 cells treated with cGAMP or primary mouse embryonic fibroblasts without DSB formation [168]. Because pancreatic cells’ TBK1 activation is facilitated by ATM inhibition, IFN production is increased [169]. Additionally, TBK1 activation mediated by Mn(2+) was reduced by ATM inhibitors [170]. As a result, TBK1 may play a significant role alongside ATM in the immunological response linked to DDR (Fig. 2).

Intriguingly, a recent report indicated that cGAMP also enables it to act as the stimulator of DDR depending on STING–TBK1 but not IFN-I expression. Mechanistically, cGAS mediates the formation of cGAMP. After that, cGAMP interacts with STING to activate TANK-binding kinase 1 (TBK1). Then TBK1 elicits ATM autophosphorylation and subsequent DDR signaling, which is absent of DSBs formation in the entire process [168].

### Tumor necrosis factor (TNF) receptor-associated factor (TRAF)

When TLRs trigger TRIF activation, the tumor necrosis factor (TNF) receptor-associated factor (TRAF) reacts by escalating the NF-κB pathway and the release of inflammatory cytokines. It has been discovered that upon DNA damage, TRAF2, TRAF1, and TRAF3 all directly identify and ubiquitylate the caspase-2 dimer to start caspase-2-dependent cell death [171]. According to a different study, ATM activated TRAF6 to attract cIAP1 and translocated to the cytoplasm in a calcium-dependent way. In DSBs, NF-κB pathways were activated through the phosphorylation of TAK1 through the ATM–TRAF6–cIAP1 axis [172]. Furthermore, in a variety of cell types, TRAF4 directly binds to and stabilizes p53 in response to DNA damage [173]. TRAFs may, therefore, play a key role in how the NF-κB pathway reacts to DNA damage.

Furthermore, TRAFs play a complex role in DDR regulation as a critical player in the TNF signaling pathway and interact with numerous other proteins in varied signal transduction. For example, when genotoxic stress-induced DNA damage, TRAF2 and its partner TRAF-interacting protein with forkhead-associated domain (TIFA) enhance ubiquitination of NF-κB essential modulator (NEMO) to activate the NF-κB pathway [174]. It has been discovered that the TRAF interaction protein (TRAIP, often referred to as TTRAP, TDP2) serves as a master regulator of DNA ICL repair. The DNA replisome’s protein components are mechanically ubiquitylated by TRAIP, enabling them to enlist NEIL3 in order to break the crosslink and carry out the ensuing repair procedure. In this instance, TRAF’s interaction with TRAIP may allow it to transmit the DDR to the IFN signaling pathway [175]. Moreover, in chicken DT40 B lymphoma cells, TRAIP withstands DNA topoisomerase II-induced chromosome breakage, suggesting that TRAIP may be a therapeutic target for DNA damage-induced cancer [176]. Furthermore, when genotoxic stress is applied, TRAF family member-associated NF-κB activator (TANK) facilitates the formation of a complex with MCPIP1-USP10 to reduce TRAF6 ubiquitination, hence suppressing IL-1R/TLRs and NF-κB signaling cascade activation [177].

### NF-κB signaling cascades

The transcriptional activator of serial inflammatory cytokines, NF-κB, was engaged in a variety of cellular biological operations [178]. Thus far, two distinct forms of NF-κB signaling have been identified. Canonically, p50 (NF-κB1)/p65 (RelA) translocates into the nucleus and binds to the IFN or cytokine promoter sites. Nuclear translocation is accomplished via p52/RelB but in a noncanonical manner. The dimeric complex of NF-κB reacts to a range of stimuli, such as TLRs, RLRs, and cGAS, and transmits signals in response to the production of IFN-I and inflammatory cytokines.

Notably, genotoxic stress is prevented by activating NF-κB signaling through DNA damage, which protects cells from genotoxic chemicals [179]. It has been shown that DDR components control NF-κB signaling pathways in relation to DNA damage. To cause the NEMO to go into the cytoplasm, for instance, ATM phosphorylates it. The cytosolic NEMO degrades the NF-κB inhibitor, IκBα, by further activating the IKK complex. Thus, when DSBs happen, the NF-κB complex is released to cause IFN-α and IFN-λ production in regulating ATM [180, 181].

**Fig. 4:**
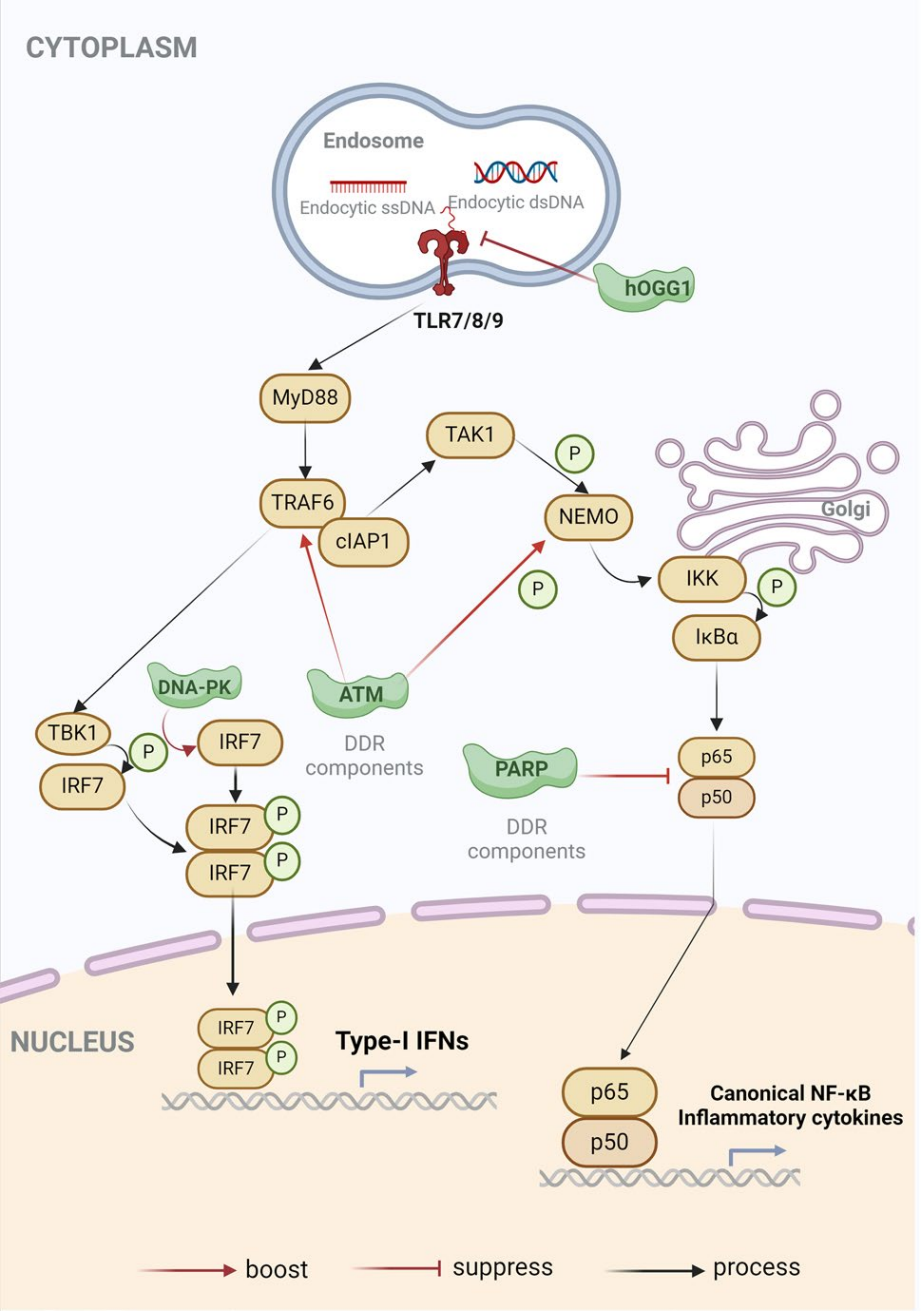
DDR components regulate NF-κB signaling pathways. Although a few DDR components have been elucidated to be involved in the NF-κB signaling pathway, the inflammation response is extensively activated in the DNA-damage repairing process. Therefore, the specific roles of DDR components in NF-κB signaling pathway may be explored in the future

However, the hyperactivation of the NF-κB pathway could lead to the overexpression of inflammatory cytokines, which would undermine hemostasis. DDR components may modulate inflammatory responses to prevent local lesions. Rat models of acute lung injury (ALI) have verified this. By blocking the NF-κB pathway, PARP inhibitors shield the ALI rat from inflammatory extravasation and prevent cell malfunction and necrosis [182]. Therefore, through interaction with the NF-κB pathway, DDR components may function as immunological modulators in some immune disorders (Fig. 4).

### IFN–IFNR–JAK–STAT signaling pathway

**Fig. 5:**
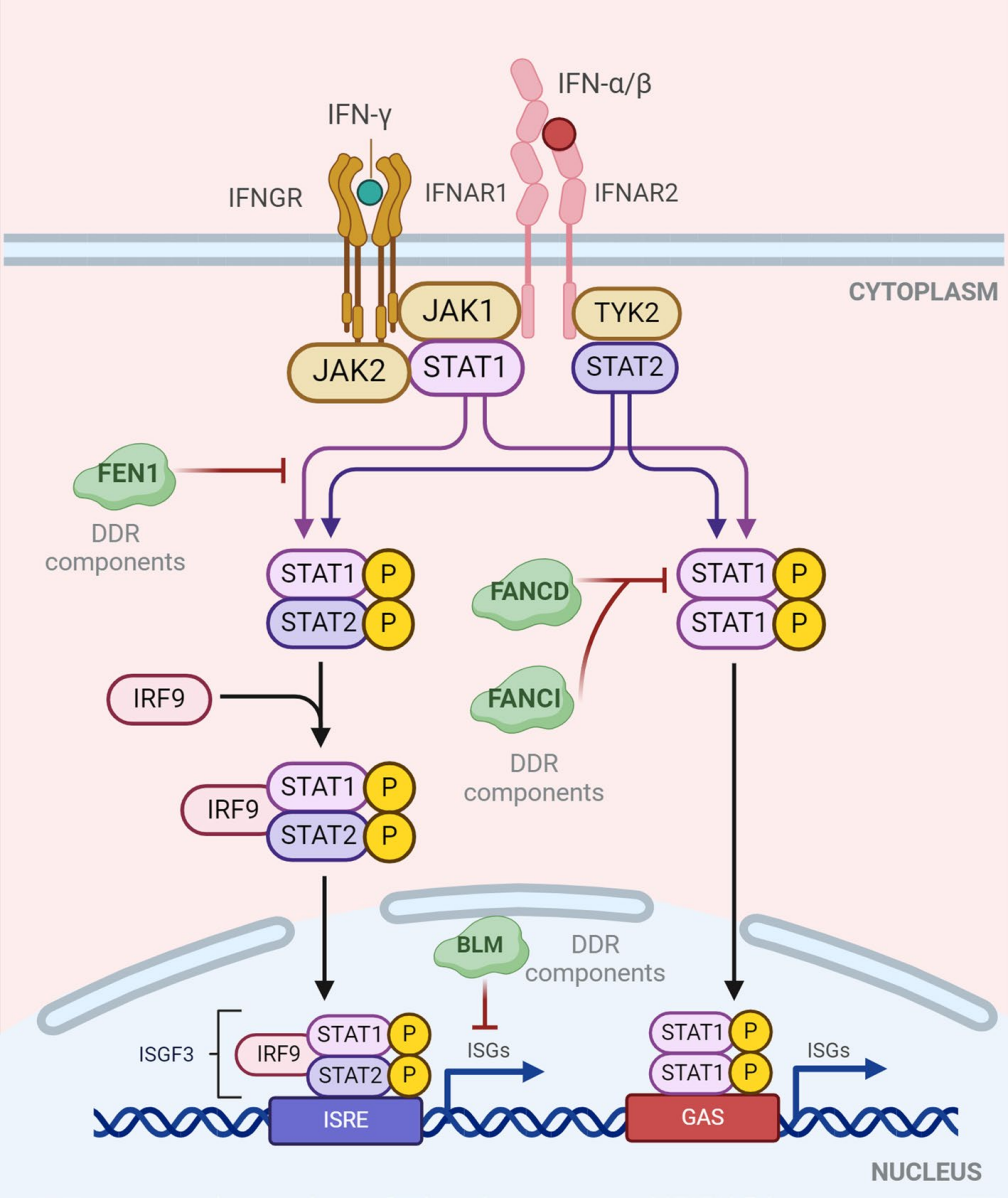
DDR components regulate JAK-STAT signaling pathways. After attachment of IFNs to its cell membrane receptors IFNAR1 or IFNAR2, the cytoplasmic domains of IFNAR1 and IFNAR2 enable the trans-phosphorylation of tyrosine kinase 2 (Tyk2, one member of JAK tyrosine kinases) and JAK1. As phosphorylases, JAK1 phosphorylates STAT1/STAT2 to facilitate them forming a heterocomplex with IRF9, the IFN-stimulated gene factor 3 (ISGF3) complex. After that, the ISGF3 translocates into the nuclei and combines the IFN-stimulated response element (ISRE) to induce ISG expression. To date, the FEN1, FANC1, BLM, or FANCD have been elucidated to participate in JAK-STAT signaling pathways. Compared to immune sensors, the JAK/STAT axis mainly functions as an effector of DNA-damage-induced immune response

The cytoplasmic domains of IFNAR1 and IFNAR2 allow the trans-phosphorylation of JAK1 and tyrosine kinase 2 (Tyk2, one member of the JAK tyrosine kinases) following the attachment of IFNs to their cell membrane receptors, IFNAR1 or IFNAR2. JAK1 and STAT1/STAT2 function as phosphorylases, allowing them to form a heterocomplex with IRF9, the IFN-stimulated gene factor 3 (ISGF3) complex. The IFN-stimulated response element (ISRE) is then combined with ISGF3 upon translocation into the nucleus to promote ISG production. It has been discovered that JAK/STAT signaling pathways, which are the distinct signaling route that reacts to IFN proteins, are involved in a number of biological processes, including DDR pathways (Fig. 5). The JAK/STAT axis primarily serves as an effector of the immunological response generated by DNA damage, in contrast to immune sensors. For instance, it has been discovered that USP1-associated factor 1 (UAF1) and ubiquitin-specific peptidase 1 (USP1) deubiquitinate the proteins FANCD2, FANCI, and PCNA to control DDR. The UAF1-USP1 association may be necessary for HCMV UL138 to control pSTAT1 for virus genomic latency, according to recent studies, raising the possibility that pSTAT may be an effector in virus infection-induced DNA damage [183].

### Interferon-stimulated genes (ISGs)

Many ISGs are actively expressed to participate in a variety of biological processes, including DDR signaling pathways, through the signal transduction of JAK–STAT. It has been discovered that ISG15, a ubiquitin-like protein, interacts with MRE11 inside the nucleus. Insufficient MRE11 causes cytosolic dsDNA buildup, which is detected by cGAS and causes the cGAS–STING signaling pathway to create IFN-I. The released IFN-Is then trigger ISG15 expression. Following that, ISG15 moves to the nuclei and becomes concentrated around DNA replication forks. Replication fork stalling, ATR activation, and genomic abnormalities are all brought on by ISG15 depletion, suggesting that ISG15 may be a regulator of the DDR process [184]. It has also been discovered that BLM-RECQL restricts the synthesis of ISG by targeting the cGAS–STING–IRF3 signaling pathway [102]. Interestingly, IFN-I also seems to reactivate cGAS expression, indicating that it may function as an ISG and complicating the relationship between DNA damage and the innate immune response (Fig. 5).

## The regulation of adaptive immune responses by DDR

As a severe detrimental process, DNA damage also systematically impacts immune cell function and surveillance, further influencing the adaptive immune response. The activation and maturation of antigen-presenting cells, such as macrophages and dendritic cells (DCs), initiate the systematic adaptive immune response. Through the conjunction of major histocompatibility complex (MHC) and T cell receptor (TCR), the naïve T or B lymphocytes are agitated to carry out cellular and humoral immunity. Compared with innate immune response, adaptive immune actions beneficially provide long-lasting protection against pathogens infection. Several investigators have delineated that DNA damage is a versatile regulator of T/B lymphocyte development and the APCs antigen-presenting process. For example, ATM, ATR, and DNA-PK are all involved in normal macrophage proliferation and differentiation. Moreover, the noncanonical DDR induced by recombination-activating gene 1 (RAG1) and RAG2-mediated DSBs has also been reviewed to regulate B-cell development and macrophage activation in various aspects [185].

### The roles of DDR in T and B cell development and function

DDR is essential to the basic development of B and T-lymphocytes. During the developing process, programmed genome alterations such as chromosomal V(D)J recombination, class-switch recombination (CSR), and somatic hyper-mutation (SHM) appear to generate diverse immunoglobulin (Ig) and T-cell receptor (TCR) [186–190].

#### The V(D)J recombination mediating TCR genes development and T cell maturation

**Fig. 6:**
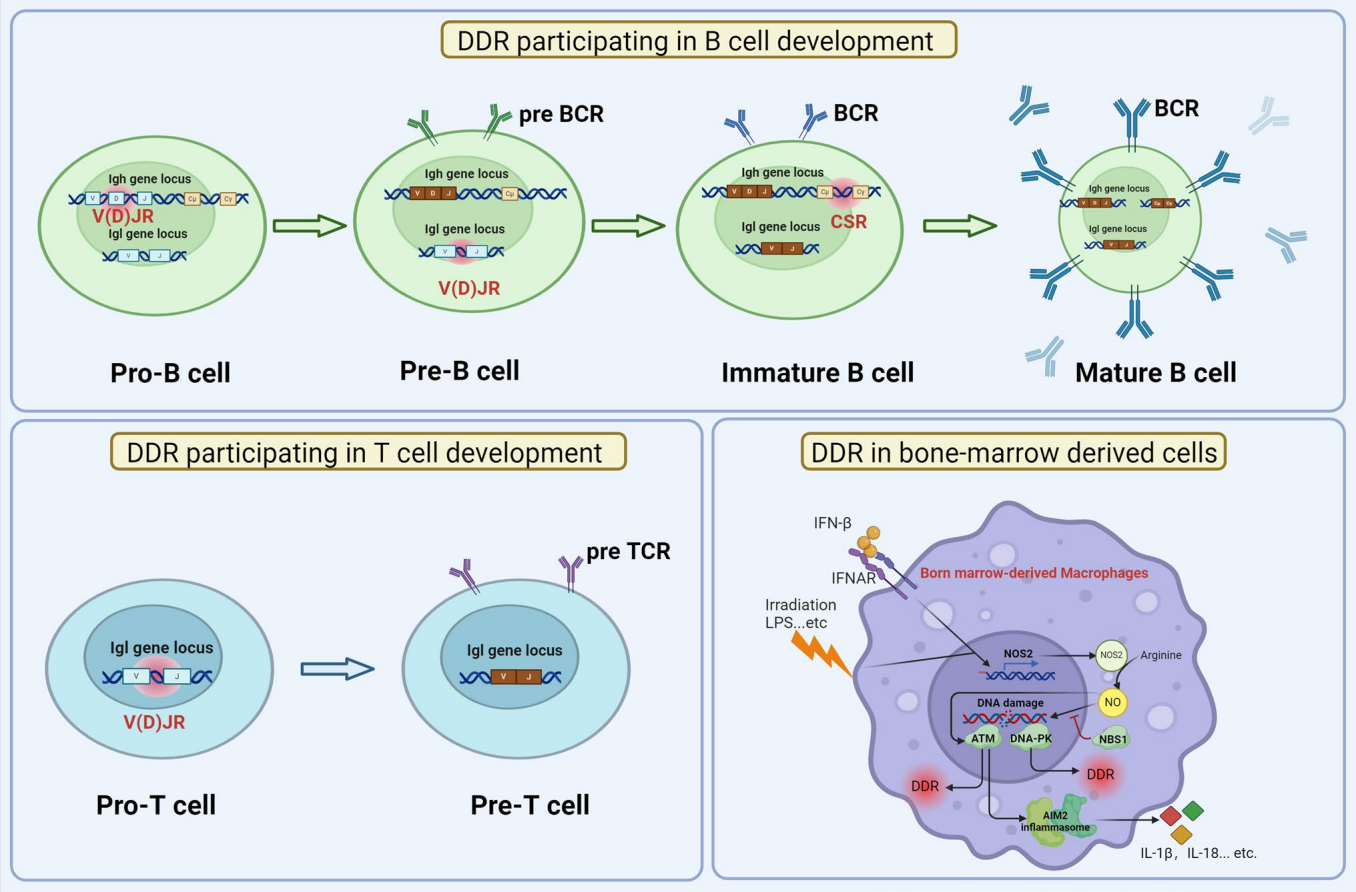
DDR participates in T/B cell development and the stimulation of bone-marrow-derived cells. (1) DDR is essential to the basic development of B and T-lymphocytes. During the developing process, programmed genome alterations such as chromosomal V(D)J recombination, class-switch recombination (CSR), and somatic hyper-mutation (SHM) appear to generate diverse immunoglobulin (Ig) and T-cell receptor (TCR). (2) DDR may also be ignited in activated macrophages due to the production of reactive oxygen intermediates (ROIs) and reactive nitrogen intermediates (e.g., nitric oxide, NO)

Among them, chromosomal V, D, and J segments in the coding regions combined to varying TCR genes by inducing DSBs, eliciting NHEJ pathways [186, 190–192]. During the recombination process, the RAG1/RAG2 protein complex recognizes the V(D)J segments and initiates the cNHEJ pathway to complete the reprogramming [186, 191, 193, 194]. Moreover, ATM, Ku complex, and XRCC/Lig 4 also functioned in the V(D)J recombination process [192]. Therefore, the DDR component mainly functions in V(D)J recombination involved in T cell maturation via regulating the TCR gene expression (Fig. 6).

#### DDR components regulate B cell maturation

The maturation of B cells is reported to require the V(D)J recombination, CSR, and SHM. Although DNA-Pkcs may have functional redundancy with other DDR apparatus in DSBs repairing during lymphocyte development [192, 195, 196], a recent study showed that mutated DNA-PKcs (five serine residues mutated to alanine residues in the S2056 cluster) in *Xlf*^*−/−*^ mice hinder the lymphocyte development, which may result from obstructive CSR efficiency [195]. Another related study indicated that DNA-PKcs phosphorylation at the T2609 cluster alters the cNHEJ to aNHEJ during the CSR process [197].

Additionally, during CSR and SHM, MMR or BER pathways are harnessed to assist the rearranging of Ig-heavy chains in the differentiation of antigen-stimulated B-cells. The latest study indicated that depletion of GEN1 and Mus81 abrogates the development and maturation of B-lineage cells or impedes the generation of robust germinal centers in early B-cell precursors and mature B cells, respectively [113]. Hence, the depletion or deficiency of DDR components yields severe failure in B and T-lymphocyte development, which further induces severe diseases such as B cell lymphomas [198–200]. Of interest, evaluating expression patterns of DDR components may prognosticate the manifestations of some lymphocytic diseases, such as chronic lymphocytic leukemia [201–203] (Fig. 6).

### The roles of DDR in the immune-stimulatory capacity of antigen-presenting cells

As professional antigen-presenting cells (APCs), dendritic cells (DCs) have been reported to intermediate the adaptive immune response induced by DNA damage. Dying tumor cells that have been treated with antitumor chemicals or irradiations might release damage-associated molecular patterns (DAMPs), which can be recognized and phagocytosis by immature DCs (iDCs) via TLRs (a process called efferocytosis) [204–206]. The iDCs then conduct the immunoproteasome activation to deliver the tumor antigens on the major histocompatibility complex (MHC) displayed on the cell surface. Pro-inflammatory cytokines, such as IL-12 [207], promote the maturation of monocyte-derived DCs (mMO-DCs) to eventually deliver the tumor antigens to CD8^+^ T cells in tumor-draining lymph nodes, which prime the formation of tumor-specific cytolytic T cells (CTLs) and the subsequent antitumor adaptive immune responses [208–210]. Additionally, depletion of IFNAR1 hindered the UVB-induced CD4^+^ T cells in mice [211]. Of interest, extending beyond mere tumor antigens, DDR components may also mediate the maturation of DCs in the context of DNA damage [156, 171]. For example, irradiation promotes the maturation of mMO-DCs through phosphorylation of NEMO, which heavily depends on ATM activation [212]. In ATM-deficient tumor cells, DNA-damage agent PBD SG-3199, resulting in IFN-I and the ISGs expression, is responsible for the maturation of DCs [62]. The cross-priming capacity of tumor-infiltrating dendritic cells (TIDC) in irradiation-treated mice depends on IFN-β autocrine [213]. Recent research demonstrated that photons, protons, and carbon ions irradiation of CD14-positive DCs have negligible effects on phenotypes, phagocytosis, migration, and IL-12 secretion capacity of both iDCs and mMO-DCs [214]. Therefore, combining irradiation and cytokines may increase the DCs maturation through immune signaling pathways in the background of DNA damage.

In addition, DDR may also be ignited in activated macrophages due to the production of reactive oxygen intermediates (ROIs) and reactive nitrogen intermediates (e.g., nitric oxide, NO). In bone marrow-derived macrophages (BMDMs), ROIs directly elicit DDR with the help of functional ATM without DSBs formation. In contrast, NO-induced DNA-PKcs-mediated DDR upon DSBs relies on IFN-I expression. However, the exact mechanisms remain unknown [146] (Fig. 6). Following the DDR activation, the key DDR components ATM or DNA-PKcs regulate a cascade of functional gene expression, including MARCO, CD69, cytokines, and chemokines (Cxcl1, Cxcl10, Ccl2, Ccl3, and Ccl4) in BMDMs [146, 185]. Interestingly, irradiation of macrophages also leads to ATM-dependent DDR [215, 216]. In this case, ATM also appears to regulate immune gene expression in injured macrophages. Other DDR components, such as NBS1 and ATR, also have been shown to play a necessary part in normal macrophage proliferation and differentiation [146, 217–220]. Furthermore, it has been demonstrated that the IFN–STAT1 pathway functions as an effector of DDR components in cancer cells by inhibiting flap endonuclease-1 (FEN1), which affects human leukocyte antigen (HLA-DR) and programmed death receptor ligand 1 (PD-L1) to suppress the tumorigenesis dependent on the IFN-γ–JAK–STAT1 pathway in oral squamous cell carcinoma [221].

## Concluding remarks

In recent years, the intricacy of the DNA damage response has been rapidly understood. The intricate relationships between DDR and immune response have come to light through various approaches, providing surprising insight into how functional DDR components operate immune signaling pathways.

Except for the immune signaling pathways, specific immune modulators may also participate in the DDR network. For example, TRIM29 has been found to be involved in modulating innate immune signaling, including IFNs [222–224], antifungal, pro-inflammatory NF-κB, and inflammasome pathways [225]. TRIM29 promotes DDR efficiency via interacting with BRCA1-associated surveillance complex and DNA-PKCs [226]. Therefore, TRM29 may also bridge the immune response to the DDR network. Additionally, PARP9 and DTX3L have recently been discovered to function as an antiviral factor in cells infected with EMCV, IAV (strain A/WS/33), or Sindbis virus (SINV) [227, 228]. Previously, PARP9 and DTX3L were identified as DDR components that form a heterodimer in DNA repair [229]. Whether PARP9 employs DDR components to regulate IFNs signaling pathways still warrants further investigations.

By controlling immunological responses, inhibitors of DDR components or deficient DDR components may function as disease-targeting therapies. There is a correlation between different types of cancer and damage to DNA. Radiotherapy, chemotherapy, and other targeted therapies are commonly utilized in clinical cancer treatments to elicit an antitumor immune response because of their ability to activate the cGAS–STING pathway by producing DNA damage. Nevertheless, this treatment approach can unintentionally cause para-carcinoma cells to sustain DNA damage, endangering surrounding healthy tissue and impeding the recovery of the patient. Furthermore, whether or not a DNA-damage targeted therapy is effective enough depends on how well it triggers the effective antitumor immune response, which has become the standard treatment for many cancers. Thus, understanding how immune response and DDR components interact will be crucial for improving anticancer immunotherapy.

In addition to cancer therapy, compromised DNA repair caused chronic inflammations, which in turn controlled autoimmune disease. For instance, in bone marrow-derived macrophages, ATM and DNA-PKcs trigger caspase 1 activation as well as the production of IL-1β and IL-18 during the creation of AIM2 inflammasomes. AIM2 inflammasome formation fails when ATM is depleted, leading to an ineffective innate immune response. Beyond DNA nuclease, other DDR components may mechanically catalyze immunological factors, which further trigger the development of inflammasomes or the release of pro-inflammatory cytokines. Therefore, targeting immune responses triggered by DNA damage or restoring DNA repair capability in autoimmune disorders could be promising options for anti-inflammatory therapy.

## Data Availability

Not applicable.

## References

[1] Valko M, Rhodes CJ, Moncol J, Izakovic M, Mazur M (2006). Free radicals, metals and antioxidants in oxidative stress-induced cancer. Chem Biol Interact.

[2] Khanna KK, Jackson SP (2001). DNA double-strand breaks: signaling, repair and the cancer connection. Nat Genet.

[3] Lindahl T (1993). Instability and decay of the primary structure of DNA. Nature.

[4] De Bont R, van Larebeke N (2004). Endogenous DNA damage in humans: a review of quantitative data. Mutagenesis.

[5] Yousefzadeh M, Henpita C, Vyas R, Soto-Palma C, Robbins P, Niedernhofer L (2021). DNA damage—how and why we age?. Elife.

[6] Harper JW, Elledge SJ (2007). The DNA damage response: ten years after. Mol Cell.

[7] Rouse J, Jackson SP (2002). Interfaces between the detection, signaling, and repair of DNA damage. Science.

[8] David SS, O'Shea VL, Kundu S (2007). Base-excision repair of oxidative DNA damage. Nature.

[9] Hoeijmakers JH (2001). Genome maintenance mechanisms for preventing cancer. Nature.

[10] Jiricny J (2006). The multifaceted mismatch-repair system. Nat Rev Mol Cell Biol.

[11] San Filippo J, Sung P, Klein H (2008). Mechanism of eukaryotic homologous recombination. Annu Rev Biochem.

[12] Lieber MR, Ma Y, Pannicke U, Schwarz K (2003). Mechanism and regulation of human non-homologous DNA end-joining. Nat Rev Mol Cell Biol.

[13] Deans AJ, West SC (2011). DNA interstrand crosslink repair and cancer. Nat Rev Cancer.

[14] Jackson SP, Bartek J (2009). The DNA-damage response in human biology and disease. Nature.

[15] Akira S, Uematsu S, Takeuchi O (2006). Pathogen recognition and innate immunity. Cell.

[16] Negishi H, Taniguchi T, Yanai H (2018). The interferon (IFN) class of cytokines and the IFN regulatory factor (IRF) transcription factor family. Cold Spring Harb Perspect Biol.

[17] Ivashkiv LB, Donlin LT (2014). Regulation of type I interferon responses. Nat Rev Immunol.

[18] Li W, Lu L, Lu J (2020). cGAS-STING-mediated DNA sensing maintains CD8(+) T cell stemness and promotes antitumor T cell therapy. Sci Transl Med.

[19] Wang X, Zhang H, Wang Y (2023). DNA sensing via the cGAS/STING pathway activates the immunoproteasome and adaptive T-cell immunity. EMBO J.

[20] Kawanishi S, Hiraku Y, Pinlaor S, Ma N (2006). Oxidative and nitrative DNA damage in animals and patients with inflammatory diseases in relation to inflammation-related carcinogenesis. Biol Chem.

[21] Boeing S, Williamson L, Encheva V (2016). Multiomic analysis of the UV-induced DNA damage response. Cell Rep.

[22] Sutherland BM, Bennett PV, Sidorkina O, Laval J (2000). Clustered DNA damages induced in isolated DNA and in human cells by low doses of ionizing radiation. Proc Natl Acad Sci USA.

[23] Barnes DE, Lindahl T (2004). Repair and genetic consequences of endogenous DNA base damage in mammalian cells. Annu Rev Genet.

[24] David SS, Williams SD (1998). Chemistry of glycosylases and endonucleases involved in base-excision repair. Chem Rev.

[25] Fromme JC, Verdine GL (2004). Base excision repair. Adv Protein Chem.

[26] Hegde ML, Hazra TK, Mitra S (2010). Functions of disordered regions in mammalian early base excision repair proteins. Cell Mol Life Sci.

[27] Lindahl T (2001). Keynote: past, present, and future aspects of base excision repair. Prog Nucleic Acid Res Mol Biol.

[28] Souliotis VL, Vlachogiannis NI, Pappa M, Argyriou A, Ntouros PA, Sfikakis pp (2019). DNA damage response and oxidative stress in systemic autoimmunity. Int J Mol Sci.

[29] Dogliotti E, Fortini P, Pascucci B, Parlanti E (2001). The mechanism of switching among multiple BER pathways. Prog Nucleic Acid Res Mol Biol.

[30] Fortini P, Parlanti E, Sidorkina OM, Laval J, Dogliotti E (1999). The type of DNA glycosylase determines the base excision repair pathway in mammalian cells. J Biol Chem.

[31] Krokan HE, Bjoras M (2013). Base excision repair. Cold Spring Harb Perspect Biol.

[32] Ding Y, Gui X, Chu X (2023). MTH1 protects platelet mitochondria from oxidative damage and regulates platelet function and thrombosis. Nat Commun.

[33] Lindahl T, Barnes DE (2000). Repair of endogenous DNA damage. Cold Spring Harb Symp Quant Biol.

[34] Lindahl T, Wood RD (1999). Quality control by DNA repair. Science.

[35] Cleaver JE, Lam ET, Revet I (2009). Disorders of nucleotide excision repair: the genetic and molecular basis of heterogeneity. Nat Rev Genet.

[36] Groisman R, Polanowska J, Kuraoka I (2003). The ubiquitin ligase activity in the DDB2 and CSA complexes is differentially regulated by the COP9 signalosome in response to DNA damage. Cell.

[37] Bregman DB, Halaban R, van Gool AJ, Henning KA, Friedberg EC, Warren SL (1996). UV-induced ubiquitination of RNA polymerase II: a novel modification deficient in cockayne syndrome cells. Proc Natl Acad Sci USA.

[38] Fanning E, Klimovich V, Nager AR (2006). A dynamic model for replication protein A (RPA) function in DNA processing pathways. Nucleic Acids Res.

[39] Thoma BS, Vasquez KM (2003). Critical DNA damage recognition functions of XPC-hHR23B and XPA-RPA in nucleotide excision repair. Mol Carcinog.

[40] Rimel JK, Taatjes DJ (2018). The essential and multifunctional TFIIH complex. Protein Sci.

[41] van Duin M, de Wit J, Odijk H (1986). Molecular characterization of the human excision repair gene ERCC-1: cDNA cloning and amino acid homology with the yeast DNA repair gene RAD10. Cell.

[42] Pascal JM, O'Brien PJ, Tomkinson AE, Ellenberger T (2004). Human DNA ligase I completely encircles and partially unwinds nicked DNA. Nature.

[43] Kastan MB, Lim DS (2000). The many substrates and functions of ATM. Nat Rev Mol Cell Biol.

[44] Bonilla B, Hengel SR, Grundy MK, Bernstein KA (2020). RAD51 gene family structure and function. Annu Rev Genet.

[45] Wright WD, Heyer WD (2014). Rad54 functions as a heteroduplex DNA pump modulated by its DNA substrates and Rad51 during D loop formation. Mol Cell.

[46] Kwon Y, Rosner H, Zhao W (2023). DNA binding and RAD51 engagement by the BRCA2 C-terminus orchestrate DNA repair and replication fork preservation. Nat Commun.

[47] Wojnicki K, Kaczmarczyk A, Wojtas B, Kaminska B (2023). BLM helicase overexpressed in human gliomas contributes to diverse responses of human glioma cells to chemotherapy. Cell Death Discov.

[48] Ubersax JA, Woodbury EL, Quang PN (2003). Targets of the cyclin-dependent kinase Cdk1. Nature.

[49] Panier S, Boulton SJ (2014). Double-strand break repair: 53BP1 comes into focus. Nat Rev Mol Cell Biol.

[50] Prakash R, Zhang Y, Feng W, Jasin M (2015). Homologous recombination and human health: the roles of BRCA1, BRCA2, and associated proteins. Cold Spring Harb Perspect Biol.

[51] Squatrito M, Gorrini C, Amati B (2006). Tip60 in DNA damage response and growth control: many tricks in one HAT. Trends Cell Biol.

[52] Johnson N, Cai D, Kennedy RD (2009). Cdk1 participates in BRCA1-dependent S phase checkpoint control in response to DNA damage. Mol Cell.

[53] Chen X, Xu X, Chen Y (2021). Structure of an activated DNA-PK and its implications for NHEJ. Mol Cell.

[54] Junop MS, Modesti M, Guarne A, Ghirlando R, Gellert M, Yang W (2000). Crystal structure of the Xrcc4 DNA repair protein and implications for end joining. EMBO J.

[55] Sibanda BL, Critchlow SE, Begun J (2001). Crystal structure of an Xrcc4-DNA ligase IV complex. Nat Struct Biol.

[56] Deriano L, Roth DB (2013). Modernizing the nonhomologous end-joining repertoire: alternative and classical NHEJ share the stage. Annu Rev Genet.

[57] Kottemann MC, Smogorzewska A (2013). Fanconi anaemia and the repair of watson and crick DNA crosslinks. Nature.

[58] Boddy MN, Gaillard PHL, McDonald WH, Shanahan P, Yates JR, Russell P (2001). Mus81-Eme1 are essential components of a holliday junction resolvase. Cell.

[59] Wood RD (2010). Mammalian nucleotide excision repair proteins and interstrand crosslink repair. Environ Mol Mutagen.

[60] Bai P, Fan T, Sun G, Wang X, Zhao L, Zhong R (2023). The dual role of DNA repair protein MGMT in cancer prevention and treatment. DNA Repair.

[61] Blackford AN, Jackson SP (2017). ATM, ATR, and DNA-PK: the trinity at the heart of the DNA damage response. Mol Cell.

[62] Lopez-Pelaez M, Young L, Vazquez-Chantada M (2022). Targeting DNA damage response components induces enhanced STING-dependent type-I IFN response in ATM deficient cancer cells and drives dendritic cell activation. Oncoimmunology.

[63] Gao Y, Li Y, Lin Z (2023). Ataxia telangiectasia mutated kinase inhibition promotes irradiation-induced PD-L1 expression in tumour-associated macrophages through IFN-I/JAK signalling pathway. Immunology.

[64] Wang L, Yang L, Wang C (2020). Inhibition of the ATM/Chk2 axis promotes cGAS/STING signaling in ARID1A-deficient tumors. J Clin Invest.

[65] Manolakou T, Nikolopoulos D, Gkikas D (2022). ATR-mediated DNA damage responses underlie aberrant B cell activity in systemic lupus erythematosus. Sci Adv.

[66] Feng X, Tubbs A, Zhang C (2020). ATR inhibition potentiates ionizing radiation-induced interferon response via cytosolic nucleic acid-sensing pathways. EMBO J.

[67] Vendetti FP, Pandya P, Clump DA (2023). The schedule of ATR inhibitor AZD6738 can potentiate or abolish antitumor immune responses to radiotherapy. JCI Insight.

[68] Lloyd RL, Wijnhoven PWG, Ramos-Montoya A (2020). Combined PARP and ATR inhibition potentiates genome instability and cell death in ATM-deficient cancer cells. Oncogene.

[69] Hopfner KP, Hornung V (2020). Molecular mechanisms and cellular functions of cGAS-STING signalling. Nat Rev Mol Cell Biol.

[70] Ablasser A, Chen ZJ (2019). cGAS in action: expanding roles in immunity and inflammation. Science.

[71] Li XD, Wu J, Gao D, Wang H, Sun L, Chen ZJ (2013). Pivotal roles of cGAS-cGAMP signaling in antiviral defense and immune adjuvant effects. Science.

[72] Chen Q, Sun L, Chen ZJ (2016). Regulation and function of the cGAS-STING pathway of cytosolic DNA sensing. Nat Immunol.

[73] Michalski S, de Oliveira Mann CC, Stafford CA (2020). Structural basis for sequestration and autoinhibition of cGAS by chromatin. Nature.

[74] Pathare GR, Decout A, Gluck S (2020). Structural mechanism of cGAS inhibition by the nucleosome. Nature.

[75] Zhao B, Xu P, Rowlett CM (2020). The molecular basis of tight nuclear tethering and inactivation of cGAS. Nature.

[76] Liu H, Zhang H, Wu X (2018). Nuclear cGAS suppresses DNA repair and promotes tumorigenesis. Nature.

[77] Jiang H, Xue X, Panda S (2019). Chromatin-bound cGAS is an inhibitor of DNA repair and hence accelerates genome destabilization and cell death. EMBO J.

[78] Zhu H, Zheng C (2021). When PARPs meet antiviral innate immunity. Trends Microbiol.

[79] Ho SS, Zhang WY, Tan NY (2016). The DNA structure-specific endonuclease MUS81 mediates DNA sensor STING-dependent host rejection of prostate cancer cells. Immunity.

[80] Ma H, Kang Z, Foo TK, Shen Z, Xia B (2023). Disrupted BRCA1-PALB2 interaction induces tumor immunosuppression and T-lymphocyte infiltration in HCC through cGAS-STING pathway. Hepatology.

[81] Schubert N, Schumann T, Daum E (2022). Genome replication is associated with release of immunogenic DNA waste. Front Immunol.

[82] Crow YJ, Hayward BE, Parmar R (2006). Mutations in the gene encoding the 3′–5′ DNA exonuclease TREX1 cause aicardi-goutieres syndrome at the AGS1 locus. Nat Genet.

[83] Guan J, Lu C, Jin Q (2021). MLH1 Deficiency-triggered DNA hyperexcision by exonuclease 1 activates the cGAS-STING pathway. Cancer Cell.

[84] Wolf C, Rapp A, Berndt N (2016). RPA and Rad51 constitute a cell intrinsic mechanism to protect the cytosol from self DNA. Nat Commun.

[85] Li J, Ko JM, Dai W (2021). Depletion of DNA polymerase theta inhibits tumor growth and promotes genome instability through the cGAS-STING-ISG pathway in esophageal squamous cell carcinoma. Cancers.

[86] Coquel F, Silva MJ, Techer H (2018). SAMHD1 acts at stalled replication forks to prevent interferon induction. Nature.

[87] Martinez-Lopez A, Martin-Fernandez M, Buta S, Kim B, Bogunovic D, Diaz-Griffero F (2018). SAMHD1 deficient human monocytes autonomously trigger type I interferon. Mol Immunol.

[88] Crossley MP, Song C, Bocek MJ (2023). R-loop-derived cytoplasmic RNA-DNA hybrids activate an immune response. Nature.

[89] Chen M, Yu S, van der Sluis T (2024). cGAS-STING pathway expression correlates with genomic instability and immune cell infiltration in breast cancer. NPJ Breast Cancer.

[90] Patterson-Fortin J, Jadhav H, Pantelidou C (2023). Polymerase theta inhibition activates the cGAS-STING pathway and cooperates with immune checkpoint blockade in models of BRCA-deficient cancer. Nat Commun.

[91] Wang J, Kang L, Song D (2017). Ku70 senses HTLV-1 DNA and modulates HTLV-1 replication. J Immunol.

[92] Weitzman MD, Lilley CE, Chaurushiya MS (2010). Genomes in conflict: maintaining genome integrity during virus infection. Annu Rev Microbiol.

[93] Kim T, Kim TY, Song YH, Min IM, Yim J, Kim TK (1999). Activation of interferon regulatory factor 3 in response to DNA-damaging agents. J Biol Chem.

[94] Karpova AY, Trost M, Murray JM, Cantley LC, Howley PM (2002). Interferon regulatory factor-3 is an in vivo target of DNA-PK. Proc Natl Acad Sci USA.

[95] Brzostek-Racine S, Gordon C, Van Scoy S, Reich NC (2011). The DNA damage response induces IFN. J Immunol.

[96] Gusho E, Laimins LA (2022). Human papillomaviruses sensitize cells to DNA damage induced apoptosis by targeting the innate immune sensor cGAS. PLoS Pathog.

[97] Jahun AS, Sorgeloos F, Chaudhry Y (2023). Leaked genomic and mitochondrial DNA contribute to the host response to noroviruses in a STING-dependent manner. Cell Rep.

[98] Harding SM, Benci JL, Irianto J, Discher DE, Minn AJ, Greenberg RA (2017). Mitotic progression following DNA damage enables pattern recognition within micronuclei. Nature.

[99] Mackenzie KJ, Carroll P, Martin CA (2017). cGAS surveillance of micronuclei links genome instability to innate immunity. Nature.

[100] Heyza JR, Ekinci E, Lindquist J (2023). ATR inhibition overcomes platinum tolerance associated with ERCC1- and p53-deficiency by inducing replication catastrophe. NAR Cancer.

[101] Schoonen PM, Kok YP, Wierenga E (2019). Premature mitotic entry induced by ATR inhibition potentiates olaparib inhibition-mediated genomic instability, inflammatory signaling, and cytotoxicity in BRCA2-deficient cancer cells. Mol Oncol.

[102] Gratia M, Rodero MP, Conrad C (2019). Bloom syndrome protein restrains innate immune sensing of micronuclei by cGAS. J Exp Med.

[103] Reislander T, Lombardi EP, Groelly FJ (2019). BRCA2 abrogation triggers innate immune responses potentiated by treatment with PARP inhibitors. Nat Commun.

[104] Oh G, Wang A, Wang L (2023). POLQ inhibition elicits an immune response in homologous recombination-deficient pancreatic adenocarcinoma via cGAS/STING signaling. J Clin Invest.

[105] Flynn PJ, Koch PD, Mitchison TJ (2021). Chromatin bridges, not micronuclei, activate cGAS after drug-induced mitotic errors in human cells. Proc Natl Acad Sci USA.

[106] Woo SR, Fuertes MB, Corrales L (2014). STING-dependent cytosolic DNA sensing mediates innate immune recognition of immunogenic tumors. Immunity.

[107] Chao HH, Karagounis IV, Thomas C (2020). Combination of CHEK1/2 inhibition and ionizing radiation results in abscopal tumor response through increased micronuclei formation. Oncogene.

[108] Fang C, Mo F, Liu L (2021). Oxidized mitochondrial DNA sensing by STING signaling promotes the antitumor effect of an irradiated immunogenic cancer cell vaccine. Cell Mol Immunol.

[109] Han C, Liu Z, Zhang Y (2020). Tumor cells suppress radiation-induced immunity by hijacking caspase 9 signaling. Nat Immunol.

[110] White MJ, McArthur K, Metcalf D (2014). Apoptotic caspases suppress mtDNA-induced STING-mediated type I IFN production. Cell.

[111] West AP, Khoury-Hanold W, Staron M (2015). Mitochondrial DNA stress primes the antiviral innate immune response. Nature.

[112] Dunphy G, Flannery SM, Almine JF (2018). Non-canonical activation of the DNA sensing adaptor STING by ATM and IFI16 mediates NF-kappaB signaling after nuclear DNA damage. Mol Cell.

[113] Fernandez KC, Feeney L, Smolkin RM (2022). The structure-selective endonucleases GEN1 and MUS81 mediate complementary functions in safeguarding the genome of proliferating B lymphocytes. Elife.

[114] Stephenson AP, Schneider JA, Nelson BC (2013). Manganese-induced oxidative DNA damage in neuronal SH-SY5Y cells: attenuation of thymine base lesions by glutathione and *N*-acetylcysteine. Toxicol Lett.

[115] Oikawa S, Hirosawa I, Tada-Oikawa S, Furukawa A, Nishiura K, Kawanishi S (2006). Mechanism for manganese enhancement of dopamine-induced oxidative DNA damage and neuronal cell death. Free Radic Biol Med.

[116] Chan Wah Hak CML, Rullan A, Patin EC, Pedersen M, Melcher AA, Harrington KJ (2022). Enhancing anti-tumour innate immunity by targeting the DNA damage response and pattern recognition receptors in combination with radiotherapy. Front Oncol.

[117] Sui H, Zhou M, Imamichi H (2017). STING is an essential mediator of the Ku70-mediated production of IFN-lambda1 in response to exogenous DNA. Sci Signal.

[118] Sui H, Chen Q, Imamichi T (2021). Cytoplasmic-translocated Ku70 senses intracellular DNA and mediates interferon-lambda1 induction. Immunology.

[119] Gardai SJ, McPhillips KA, Frasch SC (2005). Cell-surface calreticulin initiates clearance of viable or apoptotic cells through trans-activation of LRP on the phagocyte. Cell.

[120] Sims GP, Rowe DC, Rietdijk ST, Herbst R, Coyle AJ (2010). HMGB1 and RAGE in inflammation and cancer. Annu Rev Immunol.

[121] Wagner H (2004). The immunobiology of the TLR9 subfamily. Trends Immunol.

[122] Kuck JL, Obiako BO, Gorodnya OM (2015). Mitochondrial DNA damage-associated molecular patterns mediate a feed-forward cycle of bacteria-induced vascular injury in perfused rat lungs. Am J Physiol Lung Cell Mol Physiol.

[123] Wang Y, Zhao X, Liu-Bryan R (2020). Role of TLR2 and TLR4 in regulation of articular chondrocyte homeostasis. Osteoarthr Cartil.

[124] Li K, Lv G, Pan L (2018). Sirt1 alleviates LPS induced inflammation of periodontal ligament fibroblasts via downregulation of TLR4. Int J Biol Macromol.

[125] Bai P, Canto C, Oudart H (2011). PARP-1 inhibition increases mitochondrial metabolism through SIRT1 activation. Cell Metab.

[126] Kauppinen A, Suuronen T, Ojala J, Kaarniranta K, Salminen A (2013). Antagonistic crosstalk between NF-kappaB and SIRT1 in the regulation of inflammation and metabolic disorders. Cell Signal.

[127] Yang Y, Liu Y, Wang Y (2022). Regulation of SIRT1 and its roles in inflammation. Front Immunol.

[128] Wu J, Zhu W, Fu H (2012). DNA-PKcs interacts with aire and regulates the expression of toll-like receptors in RAW264.7 cells. Scand J Immunol.

[129] Jiang Y, Zhu Y, Liu ZJ, Ouyang S (2017). The emerging roles of the DDX41 protein in immunity and diseases. Protein Cell.

[130] Cargill M, Venkataraman R, Lee S (2021). DEAD-Box RNA helicases and genome stability. Genes.

[131] Su C, Tang YD, Zheng C (2021). DExD/H-box helicases: multifunctional regulators in antiviral innate immunity. Cell Mol Life Sci.

[132] Li L, Germain DR, Poon HY (2016). DEAD Box 1 facilitates removal of RNA and homologous recombination at DNA double-strand breaks. Mol Cell Biol.

[133] Li L, Monckton EA, Godbout R (2008). A role for DEAD box 1 at DNA double-strand breaks. Mol Cell Biol.

[134] Frame JM, North TE (2021). Ddx41 loss R-loops in cGAS to fuel inflammatory HSPC production. Dev Cell.

[135] Smith JR, Dowling JW, McFadden MI (2023). MEF2A suppresses stress responses that trigger DDX41-dependent IFN production. Cell Rep.

[136] Zhang Z, Yuan B, Bao M, Lu N, Kim T, Liu YJ (2011). The helicase DDX41 senses intracellular DNA mediated by the adaptor STING in dendritic cells. Nat Immunol.

[137] Tan HY, Yong YK, Xue YC (2022). cGAS and DDX41-STING mediated intrinsic immunity spreads intercellularly to promote neuroinflammation in SOD1 ALS model. iScience.

[138] Takaoka A, Wang Z, Choi MK (2007). DAI (DLM-1/ZBP1) is a cytosolic DNA sensor and an activator of innate immune response. Nature.

[139] Song J, Zhang X, Yin Y (2023). Loss of RPA1 impairs peripheral T cell homeostasis and exacerbates inflammatory damage through triggering T cell necroptosis. Adv Sci.

[140] Saada J, McAuley RJ, Marcatti M, Tang TZ, Motamedi M, Szczesny B (2022). Oxidative stress induces Z-DNA-binding protein 1-dependent activation of microglia via mtDNA released from retinal pigment epithelial cells. J Biol Chem.

[141] Yang Y, Wu M, Cao D (2021). ZBP1-MLKL necroptotic signaling potentiates radiation-induced antitumor immunity via intratumoral STING pathway activation. Sci Adv.

[142] Hu B, Jin C, Li HB (2016). The DNA-sensing AIM2 inflammasome controls radiation-induced cell death and tissue injury. Science.

[143] Lammert CR, Frost EL, Bellinger CE (2020). AIM2 inflammasome surveillance of DNA damage shapes neurodevelopment. Nature.

[144] Jiang H, Swacha P, Gekara NO (2021). Nuclear AIM2-Like receptors drive genotoxic tissue injury by inhibiting DNA repair. Adv Sci.

[145] Xie B, Luo A (2022). Nucleic acid sensing pathways in DNA repair targeted cancer therapy. Front Cell Dev Biol.

[146] Morales AJ, Carrero JA, Hung PJ (2017). A type I IFN-dependent DNA damage response regulates the genetic program and inflammasome activation in macrophages. Elife.

[147] Broz P, Dixit VM (2016). Inflammasomes: mechanism of assembly, regulation and signalling. Nat Rev Immunol.

[148] Erttmann SF, Hartlova A, Sloniecka M (2016). Loss of the DNA damage repair kinase ATM impairs inflammasome-dependent anti-bacterial innate immunity. Immunity.

[149] Liu D, Lum KK, Treen N (2023). IFI16 phase separation via multi-phosphorylation drives innate immune signaling. Nucleic Acids Res.

[150] Justice JL, Kennedy MA, Hutton JE (2021). Systematic profiling of protein complex dynamics reveals DNA-PK phosphorylation of IFI16 en route to herpesvirus immunity. Sci Adv.

[151] Li T, Diner BA, Chen J, Cristea IM (2012). Acetylation modulates cellular distribution and DNA sensing ability of interferon-inducible protein IFI16. Proc Natl Acad Sci USA.

[152] Zhu H, Tang YD, Zhan G, Su C, Zheng C (2021). The critical role of PARPs in regulating innate immune responses. Front Immunol.

[153] Aglipay JA, Lee SW, Okada S (2003). A member of the pyrin family, IFI16, is a novel BRCA1-associated protein involved in the p53-mediated apoptosis pathway. Oncogene.

[154] Diner BA, Li T, Greco TM (2015). The functional interactome of PYHIN immune regulators reveals IFIX is a sensor of viral DNA. Mol Syst Biol.

[155] Howard TR, Crow MS, Greco TM, Lum KK, Li T, Cristea IM (2021). The DNA sensor IFIX drives proteome alterations to mobilize nuclear and cytoplasmic antiviral responses, with Its acetylation acting as a localization toggle. mSystems.

[156] Kondo T, Kobayashi J, Saitoh T (2013). DNA damage sensor MRE11 recognizes cytosolic double-stranded DNA and induces type I interferon by regulating STING trafficking. Proc Natl Acad Sci USA.

[157] Li Y, Wang S, Li P (2021). Rad50 promotes ovarian cancer progression through NF-kappaB activation. J Cell Mol Med.

[158] Luzwick JW, Dombi E, Boisvert RA (2021). MRE11-dependent instability in mitochondrial DNA fork protection activates a cGAS immune signaling pathway. Sci Adv.

[159] Bunke LE, Larsen CIS, Pita-Aquino JN, Jones IK, Majumder K (2023). The DNA damage sensor MRE11 regulates efficient replication of the autonomous parvovirus minute virus of mice. J Virol.

[160] Sun X, Liu T, Zhao J (2020). DNA-PK deficiency potentiates cGAS-mediated antiviral innate immunity. Nat Commun.

[161] Burleigh K, Maltbaek JH, Cambier S (2020). Human DNA-PK activates a STING-independent DNA sensing pathway. Sci Immunol.

[162] Ferguson BJ, Mansur DS, Peters NE, Ren H, Smith GL (2012). DNA-PK is a DNA sensor for IRF-3-dependent innate immunity. Elife.

[163] Liu S, Li X, Liu X, Wang J, Li L, Kong D (2022). RNA polymerase III directly participates in DNA homologous recombination. Trends Cell Biol.

[164] Jarrous N, Rouvinski A (2021). RNA polymerase III and antiviral innate immune response. Transcription.

[165] Unterholzner L (2013). The interferon response to intracellular DNA: why so many receptors?. Immunobiology.

[166] Tigano M, Vargas DC, Tremblay-Belzile S, Fu Y, Sfeir A (2021). Nuclear sensing of breaks in mitochondrial DNA enhances immune surveillance. Nature.

[167] Doke T, Mukherjee S, Mukhi D (2023). NAD(+) precursor supplementation prevents mtRNA/RIG-I-dependent inflammation during kidney injury. Nat Metab.

[168] Banerjee D, Langberg K, Abbas S (2021). A non-canonical, interferon-independent signaling activity of cGAMP triggers DNA damage response signaling. Nat Commun.

[169] Zhang Q, Green MD, Lang X (2019). Inhibition of ATM increases interferon signaling and sensitizes pancreatic cancer to immune checkpoint blockade therapy. Cancer Res.

[170] Sui H, Chen Q, Yang J, Srirattanapirom S, Imamichi T (2022). Manganese enhances DNA- or RNA-mediated innate immune response by inducing phosphorylation of TANK-binding kinase 1. iScience.

[171] Robeson AC, Lindblom KR, Wojton J, Kornbluth S, Matsuura K (2018). Dimer-specific immunoprecipitation of active caspase-2 identifies TRAF proteins as novel activators. EMBO J.

[172] Hinz M, Stilmann M, Arslan SC, Khanna KK, Dittmar G, Scheidereit C (2010). A cytoplasmic ATM-TRAF6-cIAP1 module links nuclear DNA damage signaling to ubiquitin-mediated NF-kappaB activation. Mol Cell.

[173] Sax JK, El-Deiry WS (2003). Identification and characterization of the cytoplasmic protein TRAF4 as a p53-regulated proapoptotic gene. J Biol Chem.

[174] Fu J, Huang D, Yuan F (2018). TRAF-interacting protein with forkhead-associated domain (TIFA) transduces DNA damage-induced activation of NF-kappaB. J Biol Chem.

[175] Wu RA, Semlow DR, Kamimae-Lanning AN (2019). TRAIP is a master regulator of DNA interstrand crosslink repair. Nature.

[176] Zeng Z, Cortes-Ledesma F, El Khamisy SF, Caldecott KW (2011). TDP2/TTRAP is the major 5′-tyrosyl DNA phosphodiesterase activity in vertebrate cells and is critical for cellular resistance to topoisomerase II-induced DNA damage. J Biol Chem.

[177] Wang W, Huang X, Xin HB, Fu M, Xue A, Wu ZH (2015). TRAF family member-associated NF-kappaB activator (TANK) inhibits genotoxic nuclear factor kappaB activation by facilitating deubiquitinase USP10-dependent deubiquitination of TRAF6 ligase. J Biol Chem.

[178] Liu T, Zhang L, Joo D, Sun SC (2017). NF-kappaB signaling in inflammation. Signal Transduct Target Ther.

[179] Oh S, Bournique E, Bowen D (2021). Genotoxic stress and viral infection induce transient expression of APOBEC3A and pro-inflammatory genes through two distinct pathways. Nat Commun.

[180] Wu ZH, Shi Y, Tibbetts RS, Miyamoto S (2006). Molecular linkage between the kinase ATM and NF-kappaB signaling in response to genotoxic stimuli. Science.

[181] Wu ZH, Wong ET, Shi Y (2010). ATM- and NEMO-dependent ELKS ubiquitination coordinates TAK1-mediated IKK activation in response to genotoxic stress. Mol Cell.

[182] Martins V, Santos SS, Rodrigues L, Salomao R, Liaudet L, Szabo C (2022). Efficacy of clinically used PARP inhibitors in a murine model of acute lung injury. Cells.

[183] Zarrella K, Longmire P, Zeltzer S (2023). Human cytomegalovirus UL138 interaction with USP1 activates STAT1 in infection. PLoS Pathog.

[184] Wardlaw CP, Petrini JHJ (2022). ISG15 conjugation to proteins on nascent DNA mitigates DNA replication stress. Nat Commun.

[185] Bednarski JJ, Sleckman BP (2019). At the intersection of DNA damage and immune responses. Nat Rev Immunol.

[186] Bednarski JJ, Sleckman BP (2012). Lymphocyte development: integration of DNA damage response signaling. Adv Immunol.

[187] Bassing CH, Swat W, Alt FW (2002). The mechanism and regulation of chromosomal V(D)J recombination. Cell.

[188] Schatz DG, Swanson PC (2011). V(D)J recombination: mechanisms of initiation. Annu Rev Genet.

[189] Alt FW, Zhang Y, Meng FL, Guo C, Schwer B (2013). Mechanisms of programmed DNA lesions and genomic instability in the immune system. Cell.

[190] Boboila C, Alt FW, Schwer B (2012). Classical and alternative end-joining pathways for repair of lymphocyte-specific and general DNA double-strand breaks. Adv Immunol.

[191] Desiderio S (2010). Temporal and spatial regulatory functions of the V(D)J recombinase. Semin Immunol.

[192] Kumar V, Alt FW, Oksenych V (2014). Functional overlaps between XLF and the ATM-dependent DNA double strand break response. DNA Repair.

[193] Johnston R, Mathias B, Crowley SJ (2023). Nuclease-independent functions of RAG1 direct distinct DNA damage responses in B cells. EMBO Rep.

[194] Corneo B, Wendland RL, Deriano L (2007). Rag mutations reveal robust alternative end joining. Nature.

[195] Jiang W, Estes VM, Wang XS (2019). Phosphorylation at S2053 in murine (S2056 in human) DNA-PKcs is dispensable for lymphocyte development and class switch recombination. J Immunol.

[196] Oksenych V, Kumar V, Liu X (2013). Functional redundancy between the XLF and DNA-PKcs DNA repair factors in V(D)J recombination and nonhomologous DNA end joining. Proc Natl Acad Sci USA.

[197] Crowe JL, Wang XS, Shao Z, Lee BJ, Estes VM, Zha S (2020). DNA-PKcs phosphorylation at the T2609 cluster alters the repair pathway choice during immunoglobulin class switch recombination. Proc Natl Acad Sci USA.

[198] Bassing CH, Alt FW (2004). The cellular response to general and programmed DNA double strand breaks. DNA Repair.

[199] Knittel G, Rehkamper T, Nieper P, Schmitt A, Flumann R, Reinhardt HC (2018). DNA damage pathways and B-cell lymphomagenesis. Curr Opin Hematol.

[200] Brockelmann PJ, de Jong MRW, Jachimowicz RD (2020). Targeting DNA repair, cell cycle, and tumor microenvironment in B cell lymphoma. Cells.

[201] Griffen TL, Hoff FW, Qiu Y, Burger J, Wierda W, Kornblau SM (2023). Prognostication of DNA damage response protein expression patterns in chronic lymphocytic leukemia. Int J Mol Sci.

[202] Hubner SE, de Camargo Magalhaes ES, Hoff FW (2023). DNA damage response-related proteins are prognostic for outcome in both adult and pediatric acute myelogenous leukemia patients: samples from adults and from children enrolled in a children’s oncology group study. Int J Mol Sci.

[203] Frenzel LP, Reinhardt HC, Pallasch CP (2016). Concepts of chronic lymphocytic leukemia pathogenesis: DNA damage response and tumor microenvironment. Oncol Res Treat.

[204] Banchereau J, Briere F, Caux C (2000). Immunobiology of dendritic cells. Annu Rev Immunol.

[205] Banchereau J, Steinman RM (1998). Dendritic cells and the control of immunity. Nature.

[206] Steinman RM (2012). Decisions about dendritic cells: past, present, and future. Annu Rev Immunol.

[207] Chouaib S, Chehimi J, Bani L (1994). Interleukin 12 induces the differentiation of major histocompatibility complex class I-primed cytotoxic T-lymphocyte precursors into allospecific cytotoxic effectors. Proc Natl Acad Sci USA.

[208] Gallo PM, Gallucci S (2013). The dendritic cell response to classic, emerging, and homeostatic danger signals. Implications for autoimmunity. Front Immunol.

[209] Boada-Romero E, Martinez J, Heckmann BL, Green DR (2020). The clearance of dead cells by efferocytosis. Nat Rev Mol Cell Biol.

[210] Ghiringhelli F, Apetoh L, Tesniere A (2009). Activation of the NLRP3 inflammasome in dendritic cells induces IL-1beta-dependent adaptive immunity against tumors. Nat Med.

[211] Sherwani MA, Ahmad I, Lewis MJ (2022). Type I interferons enhance the repair of ultraviolet radiation-induced DNA damage and regulate cutaneous immune suppression. Int J Mol Sci.

[212] Parker JJ, Jones JC, Strober S, Knox SJ (2013). Characterization of direct radiation-induced immune function and molecular signaling changes in an antigen presenting cell line. Clin Immunol.

[213] Burnette BC, Liang H, Lee Y (2011). The efficacy of radiotherapy relies upon induction of type i interferon-dependent innate and adaptive immunity. Cancer Res.

[214] Konig L, Hommertgen A, Orschiedt L (2022). Influence of photon, proton and carbon ion irradiation on differentiation, maturation and functionality of dendritic cells. Front Biosci (Schol Ed).

[215] Purbey PK, Scumpia PO, Kim PJ (2017). Defined sensing mechanisms and signaling pathways contribute to the global inflammatory gene expression output elicited by ionizing radiation. Immunity.

[216] Teresa Pinto A, Laranjeiro Pinto M, Patricia Cardoso A (2016). Ionizing radiation modulates human macrophages towards a pro-inflammatory phenotype preserving their pro-invasive and pro-angiogenic capacities. Sci Rep.

[217] Pereira-Lopes S, Tur J, Calatayud-Subias JA, Lloberas J, Stracker TH, Celada A (2015). NBS1 is required for macrophage homeostasis and functional activity in mice. Blood.

[218] Herrtwich L, Nanda I, Evangelou K (2018). DNA damage signaling instructs polyploid macrophage fate in granulomas. Cell.

[219] Herrtwich L, Nanda I, Evangelou K (2016). DNA damage signaling instructs polyploid macrophage fate in granulomas. Cell.

[220] Ciccia A, Elledge SJ (2010). The DNA damage response: making it safe to play with knives. Mol Cell.

[221] Wang S, Wang X, Sun J (2023). Down-regulation of DNA key protein-FEN1 inhibits OSCC growth by affecting immunosuppressive phenotypes via IFN-gamma/JAK/STAT-1. Int J Oral Sci.

[222] Xing J, Zhang A, Zhang H (2017). TRIM29 promotes DNA virus infections by inhibiting innate immune response. Nat Commun.

[223] Liu J, Cao X (2016). Cellular and molecular regulation of innate inflammatory responses. Cell Mol Immunol.

[224] Xing J, Weng L, Yuan B (2016). Identification of a role for TRIM29 in the control of innate immunity in the respiratory tract. Nat Immunol.

[225] Xu X, Qin Z, Zhang C (2023). TRIM29 promotes podocyte pyroptosis in diabetic nephropathy through the NF-kB/NLRP3 inflammasome pathway. Cell Biol Int.

[226] Masuda Y, Takahashi H, Sato S (2015). TRIM29 regulates the assembly of DNA repair proteins into damaged chromatin. Nat Commun.

[227] Zhang Y, Mao D, Roswit WT (2015). PARP9-DTX3L ubiquitin ligase targets host histone H2BJ and viral 3C protease to enhance interferon signaling and control viral infection. Nat Immunol.

[228] Xing J, Zhang A, Du Y (2021). Identification of poly(ADP-ribose) polymerase 9 (PARP9) as a noncanonical sensor for RNA virus in dendritic cells. Nat Commun.

[229] Yan Q, Xu R, Zhu L (2013). BAL1 and its partner E3 ligase, BBAP, link Poly(ADP-ribose) activation, ubiquitylation, and double-strand DNA repair independent of ATM, MDC1, and RNF8. Mol Cell Biol.

